# SF3B1 mutant MDS-initiating cells may arise from the haematopoietic stem cell compartment

**DOI:** 10.1038/ncomms10004

**Published:** 2015-12-08

**Authors:** Syed A. Mian, Kevin Rouault-Pierre, Alexander E. Smith, Thomas Seidl, Irene Pizzitola, Aytug Kizilors, Austin G. Kulasekararaj, Dominique Bonnet, Ghulam J. Mufti

**Affiliations:** 1 Department of Haematological Medicine, King's College London School of Medicine, London SE5 9NU, UK; 2 Human Normal and Malignant Haematopoiesis Stem Cells and Their Microenvironment Laboratory, The Francis Crick Institute, Lincoln's Inn Fields Laboratories, London WC2A 3LY, UK; 3 Department of Haematology, King's College Hospital, London SE5 9RS, UK

## Abstract

Despite the recent evidence of the existence of myelodysplastic syndrome (MDS) stem cells in 5q-MDS patients, it is unclear whether haematopoietic stem cells (HSCs) could also be the initiating cells in other MDS subgroups. Here we demonstrate that *SF3B1* mutation(s) in our cohort of MDS patients with ring sideroblasts can arise from CD34^+^CD38^−^CD45RA^−^CD90^+^CD49f^+^ HSCs and is an initiating event in disease pathogenesis. Xenotransplantation of *SF3B1* mutant HSCs leads to persistent long-term engraftment restricted to myeloid lineage. Moreover, genetically diverse evolving subclones of mutant *SF3B1* exist in mice, indicating a branching multi-clonal as well as ancestral evolutionary paradigm. Subclonal evolution in mice is also seen in the clinical evolution in patients. Sequential sample analysis shows clonal evolution and selection of the malignant driving clone leading to AML transformation. In conclusion, our data show *SF3B1* mutations can propagate from HSCs to myeloid progeny, therefore providing a therapeutic target.

## Abstract (web summary)

Myelodysplastic syndromes (MDS) are clonal hematopoietic disorders with diverse phenotypes and can derive from hematopietic stem cells after the acquisition of specific somatic aberrations. In this study, the authors show that MDS initiating cells in some cases of sideroblastic anemia with SF3B1 mutations, can arise from hematopoietic stem cells.

## Graphical abstract

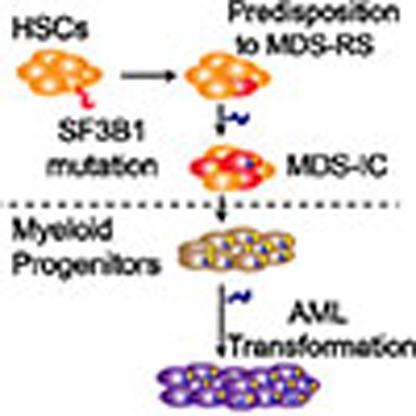

Myelodysplastic syndromes (MDS) are clonal haematopoietic disorders with diverse phenotypes, characterized by varying severity of ineffective haematopoiesis, bone marrow (BM) dysplasia, variable rates of progression to acute myeloid leukaemia (AML), overall survival and response to therapy^1^,^2^. Recent studies have implicated defects of pre-messenger RNA splicing gene *SF3B1* in the pathogenesis of MDS patients with ring sideroblasts (MDS-RS). *SF3B1* mutations are present in up to 80% of the MDS-RS patients^3^,^4^,^5^ and strongly correlate with the presence of ringed sideroblasts^4^,^5^,^6^,^7^. It is noteworthy that all the mutations reported thus far in *SF3B1* gene are heterozygous^3^,^4^,^5^,^8^, and knockout homozygous mouse models are embryonically lethal^9^.

Over the years, it has been reported that self-renewing haematopoietic stem cells (HSCs) continuously acquire somatic aberrations, while most of them are passenger mutations, some ‘potent mutations' can constitute a reservoir of pre-leukaemic stem cells^10^,^11^,^12^. The first study to report clonal spectrum at a single-cell level through multiplex fluorescence *in situ* hybridization (FISH) analysis was in childhood acute lymphoblastic leukaemia^13^. However, the recent developments of genomic technologies, stem cell isolation as well as xenotransplantation models has started to lead to a better understanding of the complex clonal architecture and mutational hierarchy of phenotypically and functionally defined ‘malignant stem cells' in AML^14^. A recent study on del(5q) MDS patients provided the first evidence of the genetic evolution and phenotypic hierarchy in del(5q) MDS before AML transformation^15^.

In MDS-RS patients, the landscape of somatic mutations has become increasingly well defined^3^,^4^,^5^,^7^,^16^. However, the specific step within the developmental schema at which a clone attains a particular genetic aberration necessary to emerge or re-emerge as a dominant clone remains unknown. For instance, we have previously shown that the sequential acquisition of oncogenic alterations (such as *FLT3* and *RUNX1*) in *SF3B1* mutant MDS-RS patients results in disease progression to AML^4^. However, the origin of *SF3B1* mutations, the detailed clonal composition (single-cell level), evolution as well as the engraftment kinetics of the haematopoietic cells that carry the *SF3B1* mutations remain unknown. Therefore, we hypothesized that *SF3B1* mutations play a central role in MDS-RS pathogenesis, can arise from the more immature HSCs and hence provide a genetic marker to study the clonal evolution from the MDS disease to leukaemic transformation.

Our data demonstrate that *SF3B1* mutations in MDS-RS patients can originate in rare HSCs and precede other known genetic lesions. Using xenotransplantation assays, we show that *SF3B1* mutant clone alone or in association with other lesions confer clonal growth advantage over ‘normal' cohabitating cells in NOD/SCID/IL2rγ^−/−^ (NSG) mice. In addition, the xenograft NSG model recapitulates the clonal changes occurring in patients' bone marrow (BM). Furthermore, the fact that *SF3B1*-mutated stem cells constitute a ‘pre-leukaemic' reservoir for more destabilizing mutations to occur points to the need of further large *in vivo* studies to identify, monitor and develop effective therapeutic strategies to prevent further subclonal evolution, recurrence and disease progression observed in MDS-RS patients.

## Results

### *SF3B1* mutations arise in HSC and persist in myeloid progeny

Whole-exome sequencing (WES) of CD34^+^ cells from a cohort of 12 MDS-RS (8 RARS, 1 RCMD-RS, 2 RARS-T and 1 tMDS; Supplementary Table 1) including 8 previously reported^4^ and 1 congenital sideroblastic anaemia patient, revealed acquired mutations in *SF3B1* in 11/13 cases (Supplementary Tables 1 and 2, Supplementary Fig. 1). A constitutional *ALAS2* (R425C) gene mutation^17^,^18^,^19^ was detected in the patient with congenital sideroblastic anaemia, but no other mutations including *SF3B1* (Supplementary Table 2) were observed in this case. Previous published studies have reported that recurrent gene mutations such as *TET2*, *DNMT3A* and *JAK2* coexist in patients with *SF3B1* mutations at variable frequencies (Supplementary Table 3)^4^,^8^,^20^,^21^. In our cohort of 12 MDS-RS patients, *SF3B1/DNMT3A*, *SF3B1/TET2, SF3B1/JAK2* coexisted in 6, 2 and 1 patient, respectively. Using WES data, we were able to use the mutant allele burden (MAB) as a mean for predicting the hierarchy of the mutations. In 2/6 cases where *DNMT3A* and *SF3B1* mutations coexist, *SF3B1* mutation was present as a dominant clone (Supplementary Fig. 1, MDS1 and -6). In 3/6 patients, *SF3B1* and *DNMT3A* mutations had similar MABs (MDS2, -10 and -12). In only one patient, *DNMT3A* (MDS11) had a *DNMT3A* clone higher than *SF3B1* (70 versus 60%, respectively).

Nevertheless, for *TET2*, and *JAK2*, where MABs between *SF3B1* and these mutations were similar, it was not possible to decipher which mutation was ancestral without further clonal analysis. Unfortunately, for MDS10, MDS11 and 12, no viable BM cells were available to further dissect the clonal composition.

Here, we used 5 out of 12 MDS-RS patients, one congenital sideroblastic anaemia and three haematologically normal controls for further *in vitro* and *in vivo* analysis (Supplementary Table 4). An additional MDS-RS patient (*n*=1; MDS14) with *SF3B1* mutation H662Q was also included in this study (Supplementary Table 1).

**Figure 1:**
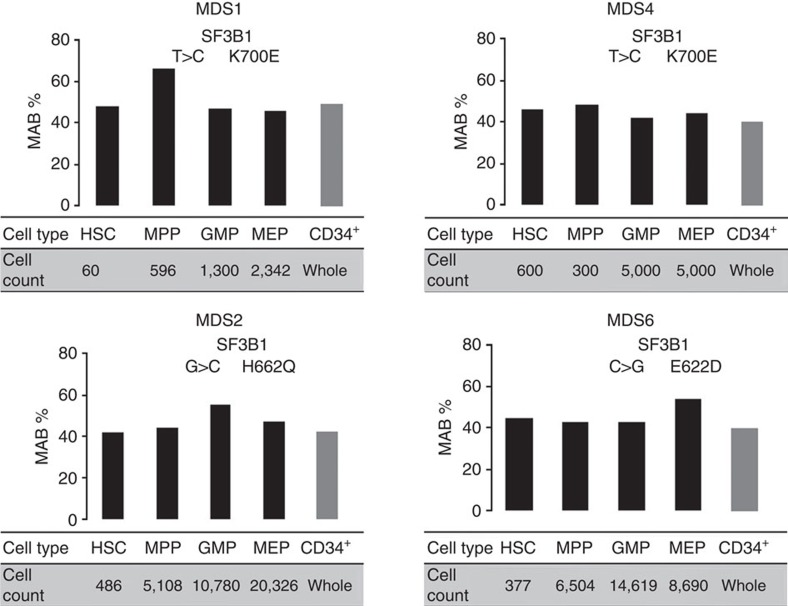
*SF3B1* mutations can propagate from HSCs to their progeny. *SF3B1* mutations occurs in rare HSC phenotypic cells, is largely maintained in more committed progenitors MPPs as well as in more differentiated progenitors GMPs and MEPs. Tables show the progenitor cell counts (events detected by the FACS during cell sorting) for each patient sample. The average sequencing coverage across all amplicons was ≥800 reads. Bar charts indicate the *SF3B1* MAB for the respective cell fraction. MAB for each cell population was confirmed by independent PCRs. MAB mutant allele burden (*t*-test *P* value>0.05).

To identify the target cell within the haematopoietic compartment, which carries the *SF3B1* mutation, HSCs, multipotent progenitors (MPPs), granulocyte macrophage progenitors (GMPs) and megakaryocyte erythrocyte progenitors (MEPs) were isolated from the BM of four patients (MDS1, MDS2, MDS4 and MDS6) harbouring *SF3B1* mutation (Supplementary Fig. 2A,B; Supplementary Table 1). To date, HSC in MDS studies have been enriched with the following markers Lin^−^CD34^+^CD38^−^CD90^+^CD45RA^−^ (ref. 22). To increase the HSCs purity, CD49f (integrin alpha 6) was used along with Lin^−^CD34^+^CD38^−^CD90^+^CD45RA^−^, which allowed us to enrich our population in long-term HSCs^23^. We performed targeted mutational analysis on the fluorescence-activated cell sorting (FACS)-purified cells from all patients and showed that *SF3B1* mutations originate in the rare HSC population (Fig. 1). *SF3B1* mutations were detected in the MPPs, GMPs and MEPs fractions in all patients (Fig. 1). Notably, the MAB was largely maintained between the HSCs, MPPs and more differentiated myeloid progenitors.

**Figure 2:**
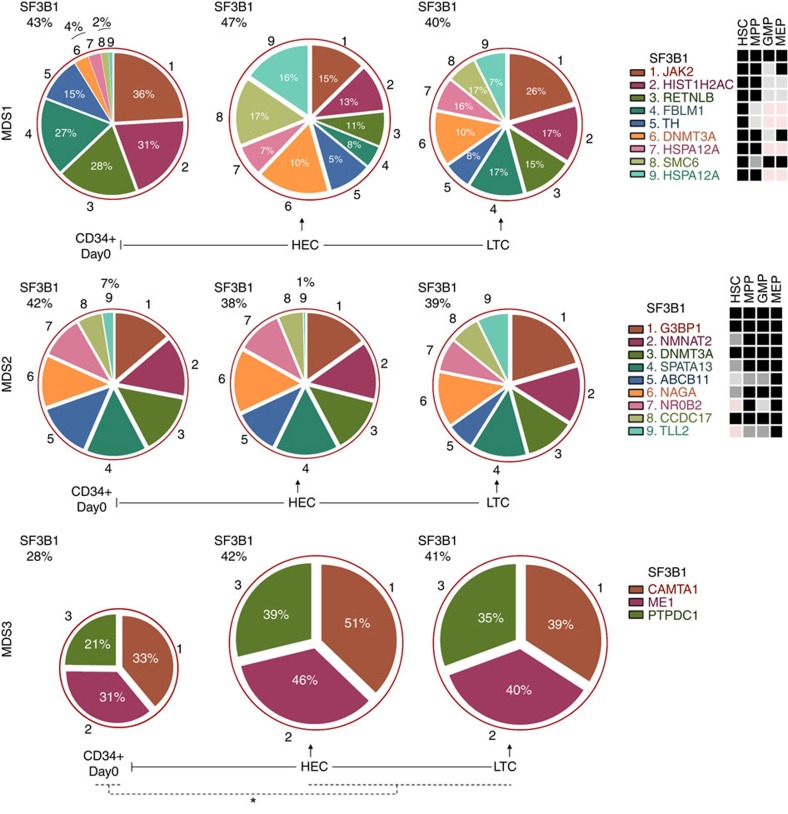
Mutational architecture and subclonal evolution of *SF3B1*-mutated MDS-RS bone marrow cells. Panels show the gene mutations with their MABs in primary CD34^+^ bone marrow, HEC cells and LTC samples from each patient. Outer red circle represents the total SF3B1 clone and the inner sections of the circle represents the SF3B1 subclones. MABs for HEC samples are the average of all mouse samples (where applicable, that is, >1 mouse). Percentages displayed in the pie chart indicate the MAB frequency modulation in HEC and LTC samples versus CD34^+^ day 0. *P<0.01% (*t*-test) for difference in SF3B1 MAB frequency between CD34^+^ day 0 and HEC and/or LTC samples for the MDS3 sample. For more details about statistical analysis of the variation in MAB frequencies, refer to Supplementary Fig. 5. The table on the right side of each panel represents the mutation status of the respective genes in the HSC, MPP, GMP and MEP cell populations for each patient. Samples used to isolate cell fractions (HSCs, MPPs, GMPs and MEPs) were taken ∼18 months later than the first sample used to isolate CD34^+^ cells. Three independent PCRs were performed to confirm/determine the MAB throughout the experiments. In the tables on the right side, the black boxes indicate that MAB for gene mutations was the same as the primary CD34^+^ cells, dark grey boxes show MAB for gene mutations that was≤2-fold as compared to the primary CD34^+^ cells, light grey boxes represent MAB for gene mutations that was ≥2-fold as compared with the primary CD34^+^ cells and pink boxes indicate not detected or below background noise level. MAB mutant allele burden; HEC, human engrafted cell; LTC, long-term culture; BM, bone marrow; Mut, mutation.

WES data from three of the patients (MDS1, MDS2 and MDS4) identified additional patient-specific mutations in CD34^+^ cells. Next, we sought to determine whether these patient-specific mutations originate from the same HSC malignant pool as *SF3B1* or whether they occur downstream of the stem cell differentiation in MPPs, GMPs and MEPs. Mutational analysis demonstrated that the majority of the patient-specific mutations including clinically important myeloid-specific genes such as *DNMT3A* and *JAK2* were present in the rare HSC population and in the mature myeloid progenitors, that is, MPPs, GMPs and MEPs (Fig. 2). We also observed in some cases that ‘patient-specific mutations' are either gained or lost at the mature progenitor level. This could be due to the fact that these mutations are present as minor clones (<10% MAB) and are as such diluted further. None of the gene mutations including *SF3B1* were detected in the mesenchymal stromal cells cultured from the patient's BM samples (data not shown). Furthermore, we and others have previously shown that *SF3B1* mutations are not detected in T- or B cells in MDS-RS patients^4^,^5^. Taken together, in our cohort of patients *SF3B1* mutations are clonally propagated by HSCs to their mature myeloid progenitors, but not to the lymphoid progenitors.

### MDS-ICs are restricted to the rare HSC fraction

**Figure 3:**
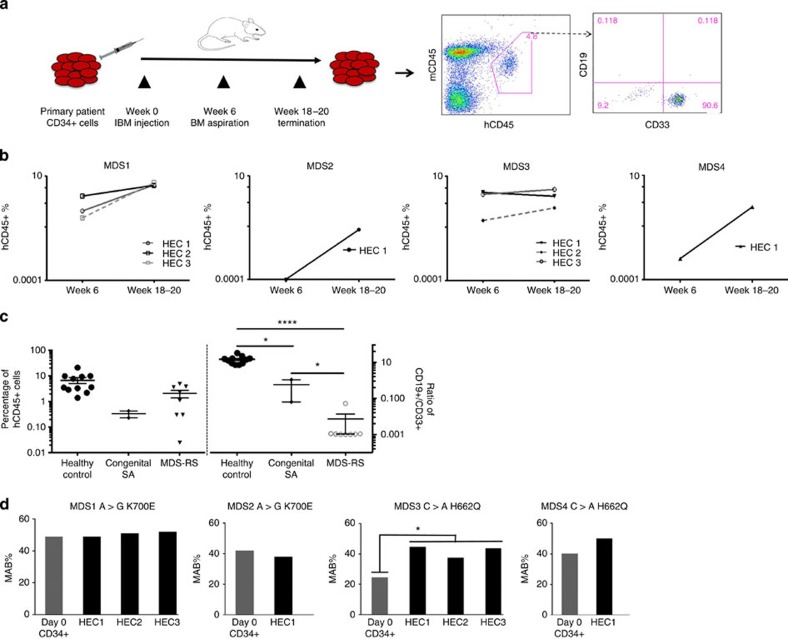
Engraftment and genotype of human RARS-originated and control haematopoietic cells in the bone marrow of NOD/SCID/IL2rγ^−/−^ mice. (**a**) Schematic representation of the xenograft model. Right-hand side panel shows the flow cytometry profile of bone marrow cells recovered from one human-cell-engrafted mice. The majority of human CD45-expressing cells were positive for a myeloid marker CD33^+^ in all analysed cases in this study. (**b**) Percentage of human CD45^+^ cells in the bone marrow of NSG mice at 6 and 18–20 weeks after transplantation (MDS1, *n*=3; MDS2, *n*=1; MDS3, *n*=3 and MDS4, *n*=1). (**c**) Percentages of human haematopoietic cells and ratio of CD19^+^/CD33^+^ isolated from the bone marrow of mice engrafted with either MDS-RS (*n*=4 patients and transplanted in a total eight mice) or healthy controls CD34^+^ cells (*n*=3 healthy donors and transplanted in a total of 11 mice) or congenital sideroblastic anaemia patient (*n*=1 patient transplanted in two mice). The *y* axis (left) represents the percentage of the human CD45^+^ cells present in the total mouse bone marrow. The *y* axis (right) represents the ratio of the human CD19^+^ versus human CD33^+^ cells within the human CD45^+^ cells recovered following xenotransplant. MDS xenografts showed a significant skewing towards myeloid lineage. *P<0.05, ****P<0.0001 (*t*-test). (**d**) Targeted mutational analysis shows the presence of concordant *SF3B1* mutations in primary CD34^+^ bone marrow sample (grey) and xenograft (black) in all analysed cases. Three independent PCR/sequencing experiments were performed to confirm/determine the mutant allele burden throughout the experiments. The sequencing coverage across the *SF3B1* amplicons was ≥1,000 reads. *P<0.01 (*t*-test).

The NSG mice models in which primary human leukaemic cells are transplanted have previously been used to expand our understanding of the pathogenesis of human leukaemia^13^,^24^. Although MDS patient cells are difficult to engraft in xenograft models, the recent use of NSG mice as well as intra-bone injection (with or without co-injection of mesenchymal stroma cells) have been reported to yield higher engraftment^15^,^22^,^25^. Therefore, we prepared CD34^*+*^ BM cells from four *SF3B1*-mutated MDS-RS patients (MDS1, TP1; MDS2, TP1; MDS3; MDS4) and one congenital sideroblastic anaemia patient (MDS5 with *ALAS1* mutation), which was used as a control. Following on, these cells were then transplanted into NSG mice (Fig. 3a; Supplementary Table 4). In parallel, we injected adult BM CD34^*+*^ cells from three healthy donors into NSG mice to control any lineage bias.

Engraftment levels based on human CD45^+^ cells harvested from engrafted mice at week 18–20 ranged between 0.1 and 4.9% (Fig. 3b; Supplementary Table 4). Majority of the cells engrafted in mice were of myeloid lineage with little or no lymphoid cell engraftment. In contrast, mice injected with CD34^+^ cells from a congenital sideroblastic anaemia patient showed myeloid as well as lymphoid cell engraftment (Fig. 3c; Supplementary Table 4). Similarly, engraftment observed in mice transplanted with CD34^+^ cells from normal healthy controls exhibited a higher ratio of lymphoid/myeloid cell as compared with *SF3B1* mutant cases (Fig. 3c). It is important to emphasize that B cells in MDS-RS patients do not carry the *SF3B1* mutation^4^. Therefore, data from our study suggest that the presence of *SF3B1* mutations, perhaps along with additional ‘cooperating factors', adversely affects the lymphoid cell development in this model, which is in agreement with the previous xenotransplantation models proposed^15^,^22^.

To confirm the nature of the human engrafted CD45^+^CD33^+^ cells (HECs) recovered from mice, mutational analysis was performed on HECs (Supplementary Table 5). Interestingly, the genotypic characteristics of patients' primary CD34^+^ BM cells were maintained in HEC, as demonstrated by the presence of *SF3B1* mutations. As mentioned above, *SF3B1* mutations are heterozygous ones, therefore a MAB of 50% implies that 100% of the cells carry the mutation. *SF3B1* MAB remained largely unchanged between the primary CD34^+^ cells (≈43%), and post-transplant HEC (≈46%) in 3/4 cases (Fig. 3d). This was irrespective of the type of *SF3B1* mutations (K700E, *n*=2; H662Q, *n*=2). In one RARS patient (MDS3), *SF3B1* (K700E) MAB increased from 28% in primary CD34^+^ cells to 43% (average MAB between three mice) after xenograft transplantation. *SF3B1* mutations were also observed in cells cultured in both CFC and long-term culture (LTC) conditions, at a variable MAB frequency (Fig. 2; Supplementary Fig. 5). Therefore, our data suggest a clonal advantage of the malignant mutant clone(s) over cohabitating normal HSCs in *SF3B1* mutant MDS-RS cases.

Finally, we sought to determine which progenitor cells within the haematopoietic compartment were able to engraft in NSG mice. To address this, we isolated HSC, MPP, MLP, CMP and GMP from one MDS-RS patient (MDS14) with *SF3B1* H662Q mutation and injected each fraction separately into mice. Interestingly, the mouse that received *SF3B1*-mutated HSCs engrafted and the *SF3B1* H662Q MAB in mice was maintained (Table 1), which is similar to the recent data from Woll *et al.*^15^

### Xenograft exhibits subclonal evolution of *SF3B1* mutant clones

To further explore the clonal landscape and evolution of the *SF3B1* mutant cells in our xenograft, we performed WES and/or targeted mutational analysis on the HECs. This strategy enabled us to reliably trace *SF3B1* and coexisting mutations as well as to detect *de novo* gene mutations. All the mutations were confirmed in at least one non-whole-genome amplified (WGA) HEC sample in all cases (see Methods for more details). The degree of quantitative accuracy of the allele burdens for WGA DNA was validated independently (Supplementary Fig. 3A,B; see Methods for details).

NSG xenograft maintained (or enhanced) the malignant *SF3B1* clone in general; however, the subclonal population shift traced via the changes in the MABs was observed in 2/4 cases (Fig. 2; Supplementary Fig. 5; Supplementary Table 5). For example, in MDS1, *JAK2* (V617F) MAB decreased from 36–15% (average MAB for three mice; Fig. 2; Supplementary Fig. 5), whereas the *DNMT3A* increased to 10%. A similar trend was observed for other gene mutations in this patient. Interestingly, *DNMT3A* mutation was not initially detected in primary CD34^+^ cells by WES. However, a follow-up high-depth targeted mutational analysis confirmed the presence of these mutations at a low level (<5%) in the primary CD34^+^ cells (Fig. 2; Supplementary Fig. 5). On the other hand, *TLL2* mutation present in the MDS2 (7% MAB) CD34^+^ cells almost disappeared in the NSG mice (1% MAB, Fig. 2). In the rest of the cases, patient-specific mutations followed the same trend as *SF3B1* clones, implying the presence of a single *SF3B1* clone with multiple mutations. In all cases, similar clonal/subclonal changes were observed in pooled CFC and LTC assays.

Together our data demonstrate that *SF3B1* malignant cells were maintained or even enhanced in NSG mice and that subclonal fluctuation could be detected in certain cases. Of note, all the mutations present in the NSG mice were traced back to the patients' primary CD34^+^ cells and no new mutations were detected in the NSG mice.

### Clonal analysis reveals mutational architecture in MDS-RS

Previous studies have described mutational landscape of *SF3B1*-mutated patient BM cells^3^,^4^,^5^,^8^. However, these studies have inferred the clonal architecture in the total BM samples through the identification of clusters of coexisting mutations with similar MABs, and this does not necessarily recapitulate the actual subclonal architecture of the patients' BM compartment. Therefore, to gain insight into the clonal landscape of coexisting mutations in *SF3B1* mutant MDS-RS patients, we performed a single-cell clonogenic assay using CD34^+^ cells from four patients (MDS1, time point 1; MDS2, TP1; MDS3; MDS4), followed by targeted mutational analysis of the patient-specific mutations.

**Figure 4:**
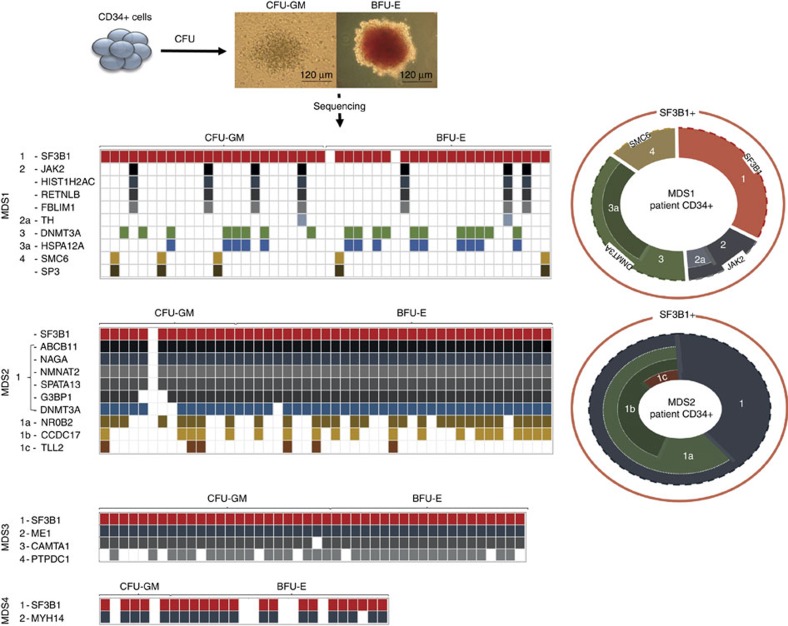
*SF3B1* mutations in single-cell colonies. Mutational analysis was performed on BFU-E and CFU-GM colonies derived from primary CD34^+^ patient cells (MDS1, MDS2, MDS3 and MDS4). Each column represents an individual CFC colony. Circle (MDS1 and MDS2) on the right is the representative of the overall clonality observed in the clonogenic assays (left) in MDS1 and MDS2 and total CD34^+^ cells. The outer red circle represents the total SF3B1 clone, while the inner circle depicts subclones. WT, wild type.

Initial mutational analysis performed on individual CFC colonies from MDS1 (48–96 colonies), MDS2 (47 colonies), MDS3 (46 colonies) and MDS4 (30 colonies) using gene-specific primers targeting *SF3B1* mutations revealed that 97%, 98%, 96% and 77% of the colonies carried *SF3B1* mutant gene, respectively (Fig. 4; Supplementary Fig. 6). Moreover, further analysis of the ‘patient-specific' gene mutations in these cases revealed the complex heterogeneity within the *SF3B1*-mutated colonies. For example, MDS1 and MDS2 had a marked subclonal heterogeneity (Fig. 4). Notably, driver mutations such as *DNMT3A* were present in 44/47 MDS2 colonies, suggesting that it may have been acquired at a later time point during disease progression. MDS1 demonstrated six different *SF3B1* subclones where 15/48 colonies had *SF3B1* mutations alone (Fig. 4; Supplementary Fig. 6). Interestingly, some of these subclones were mutually exclusive, especially subclones with known driver mutant genes such as *JAK2* and *DNMT3A*, therefore representing a branching multi-clonal evolution (Fig. 4). In contrast, the simplest of the genetic architectures was present in MDS3 and MDS4 where only two and three mutant *SF3B1* subclones were observed, respectively (Fig. 4).

It is noteworthy that CFC individual colonies with wild-type *SF3B1* (MDS1, *n*=2; MDS2, *n*=1; MDS3, *n*=2 and MDS4, *n*=7) had no additional gene mutations among those screened (Fig. 4; Supplementary Fig. 6). We therefore provide further evidence that *SF3B1* mutations are present in the majority of the CD34^+^ cells and, importantly, precede other driver mutations such as *DNMT3A* and *JAK2* as evidenced here. This implies that *SF3B1* mutation is the ‘founder mutation' in these MDS-RS patients.

### Xenograft recapitulates the clonal evolution in patients BM

**Figure 5:**
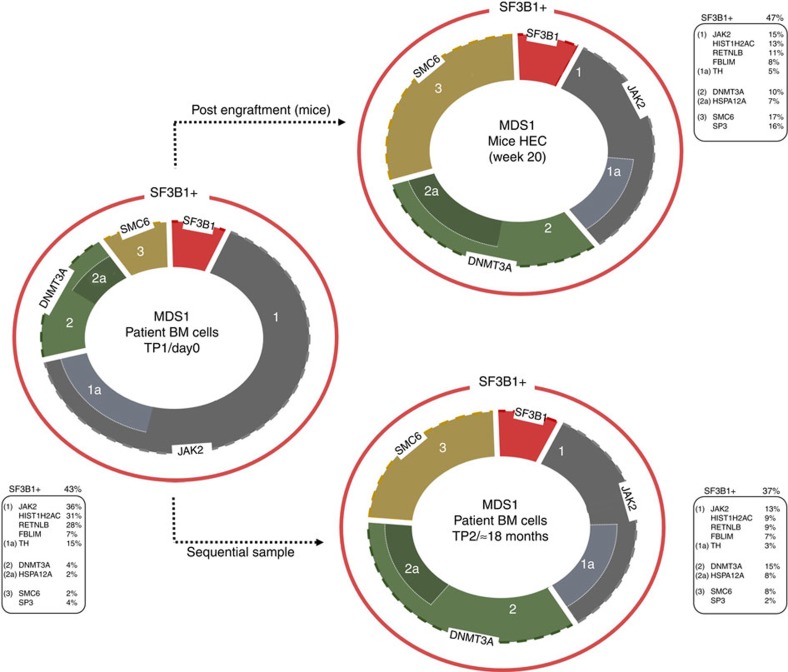
Xenograft recapitulates the clonal changes occurring in RARS patient bone marrow compartment. Mutational analysis of sequential primary TNC (total nucleated cells) sample from one patient (MDS1). Primary patient sample was received at two different time points, MDS1 bone marrow cells (TP1, first sample) and MDS1 bone marrow cells (TP2, second sample). Mutational analysis was also performed on human engrafted cells, which were obtained from mice transplanted with the patient sample received at time point 1. The length of the arc represents the MABs. Gene MAB for human engrafted cells is the average between three animal experiments. Three independent PCR/sequencing experiments were performed to confirm/determine the mutant allele burden throughout the experiments. Error Bar in the bar chart were derived from the PCR/sequencing technical replicates. MAB, mutant allele burden; TP, time point; HEC, human engrafted cells; BM, bone marrow cells.

To ascertain whether the xenograft clonal changes observed, occur in the MDS-RS patient BM, we performed targeted mutational screening on sequential samples (total nucleated cells) obtained from two patients (MDS1, ≈18 months after initial sampling with no clinical disease progression, TP2; MDS2, ≈29 months after the first sampling at the AML transformation stage, TP2; Supplementary Table 1). *SF3B1* mutations were largely unchanged between the two time points in both patients (Fig. 5; Supplementary Fig. 7). However, in MDS1, a change in the MAB of additional somatic mutations was observed in the sequential sample (TP2; Supplementary Fig. 7). For example, *JAK2* subclone (V617F, 36–13%) diminished (Fig. 5), whereas *DNMT3A* clone increased from 4–15%, in the sequential sample (TP2, Fig. 5). Strikingly, cells from TP1 engrafted into the mice and harvested at week 20, demonstrated similar subclonal changes as observed in the sequential patient TP2 (Fig. 5). These data along with our clonogenic data suggest that four *SF3B1* subclones exist in this patient; however, ‘isolated *SF3B1*' and ‘*SF3B1* plus *DNMT3A*' clones have a growth advantage over ‘*SF3B1-JAK2*' clone and others in NSG mice as well as in the human BM environment (TP2).

In MDS2, *CCDC17* and *TLL2* mutations were not detected in the sequential sample while the rest of the mutations were largely maintained. A similar trend was observed in the NSG mice where *TLL2* gene mutation diminished to 1% in the HEC sample (Supplementary Fig. 7). Altogether, our data suggest that the xenograft model does recapitulate some of the clonal changes occurring in the patients' BM in one case, while as in another case where AML transformation occurred, a partial recapitulation was observed. This could be probably due to the stochastic acquisition of additional genomic lesion at the later stage of the disease. Meanwhile, it is noteworthy that the ‘incubation period' of the MDS BM cells in NSG mice was only 20 weeks while the patient (MDS2) transformed to AML 29 months after the first MDS sampling.

### Sequential lesions in *SF3B1* clones leads to transformation

**Figure 6:**
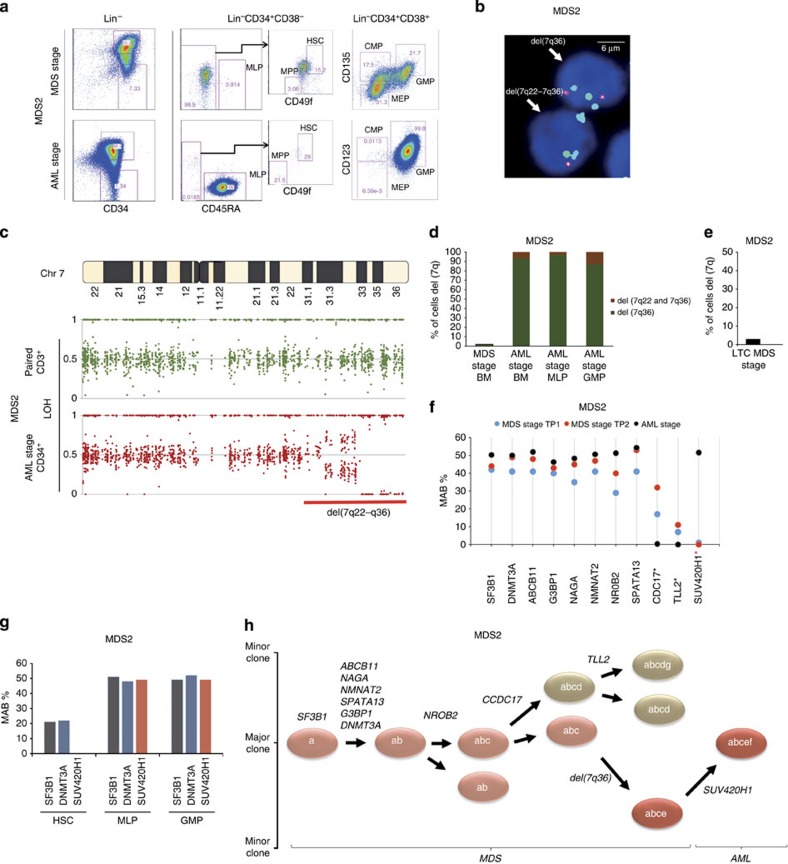
Clonal evolution from MDS to AML in RCMD-RS patient with *SF3B1* mutation. (**a**) FACS profiles of stem cell progenitors in BM MNCs in a serial sample at the MDS stage and AML stage of the disease. (**b**) Interphase FISH for tracking the del(7q) aberration in cells obtained at the AML stage of the disease. Two chromosome-7 aberrations, del(7q36) and del(7q22-7q36), were detected as two separate clones. Two individual cells are shown in the figure with one harbouring del(7q36) represented by a green probe and the other cell has del(7q22-7q36) represented by probes orange/green. (**c**) SNV profile derived from whole-exome sequencing. The shown profile depicts a heterozygous deletion (horizontal red line) of chromosome 7q in patient CD34^+^ bone marrow cells (AML stage, bottom) and absence of the deletion in paired control CD3^+^ cells (top). (**d**) Bar chart (FISH analysis) showing percentage of CD34^+^ cells (MDS stage), CD34^+^ cells (AML stage), MPL (AML stage), GMP (AML stage) with del(7q) aberration. (**e**) Bar chart (FISH analysis) showing the percentage of del(7q) aberration in LTC-derived cells from MDS-stage CD34^+^ cells (time point 1). (**f**) Mutational status of the gene mutations in sequential bone marrow samples (MDS-stage time-point 1, MDS-stage time-point 2 and AML stage). Coloured circles represent the MABs for each screened gene mutation. Black * represent absence of gene mutations in the AML-stage sample. Red * represent acquisition of additional mutation at the AML stage. (**g**) Mutational analysis of *SF3B1*, *DNMT3A* and *SUV420H1* in HSCs, MLPs and GMPs from the AML stage of the disease. Sequencing depth for HSCs was >10,000 reads. (**h**) Proposed sequential acquisition of genetic lesions based on analysis of sequential samples, LTC-derived cells and single-cell clonogenic assays.

MDS2 patient evolved into AML 29 months after the first MDS sample was taken (MDS2, TP1). It is important to note that leukaemic transformation in RARS disease is a very rare event. Initially, we performed FACS analysis on BM samples (TP3) to examine the phenotypic changes that could have occurred in the BM leading to AML (Fig. 6a). The frequency of CD34^+^ cells in BM mononuclear cells (MNCs) at AML stage (at 29 months) was significantly higher as compared with the MDS stage 35.3 and 1.54%, respectively. Interestingly, a remarkable shift of cell populations was observed within the CD34^+^CD38^−^ and CD34^+^CD38^+^ compartments showing a marked increase in MLPs (>99%) and GMPs (>99%) at the AML stage (TP3) (Fig. 6a). If this result implies the switch from a stem cell disease to a progenitor disease, where a MDS progenitor would have acquired self-renewal ability and became AML blast, we should be able to identify MDS-initiating cells (MDS-ICs) still present at the AML stage. Thus, we sought to investigate the contribution of additional genomic lesions (chromosomal aberrations; gene mutations, known ‘driver' or ‘passenger' mutations) acquired at the AML stage. Metaphase cytogenetics at the AML stage revealed complex chromosomal changes (Supplementary Table 1) including del(7q). To understand whether del(7q) was a major event in the transformation process, we traced back this chromosomal aberration by FISH analysis (Supplementary Fig. 8A) of the whole BM sample, MLPs and GMPs at the AML stage and also on the whole BM sample at the MDS stage (TP1). Two chromosome-7 aberrations were detected at the AML stage of the disease with 93% harbouring del (7q36) and 7% containing del(7q22-7q36), in the total MNCs, in the MLPs and in the GMPs (Fig. 6b,c). Interestingly, only del(7q36) was traced back to the MDS stage of the disease; however, the frequency was significantly lower, that is, 2% (Fig. 6d,e). We were unable to perform FISH analysis on the phenotypically defined HSCs, MPPs, CMPs and MEPs due to the low frequency of these progenitors in the BM at the AML stage. It is noteworthy that FISH analysis on the LTC-derived cells (MDS stage TP1) showed low-level del(7q36) clone (3%; Fig. 6d), therefore suggesting that the major clone containing del(7q36) is an early lesion acquired in the *SF3B1* mutant rare HSCs at an MDS stage of the disease. However, SNV profile through WES on LTC showed no change in the loss of heterozygosity at chromosome 7, and similar results were observed at the MDS stage for CD34^+^ cells, HEC (MDS stage) and CD3^+^ cells (Supplementary Fig. 8B).

As the del(7q) does not seem to be a ‘marker' of the AML transformation, we sought for other targets through WES. Results showed that *SF3B1* and other additional gene mutations including *DNMT3A* were maintained; however, *CCDC17* and *TLL2* were not detected (Fig. 6f). Further analysis revealed additional patient-specific mutations, including *SUV420H1* (L645F) novel mutation (Fig. 6f), which is a histone methyltransferase that specifically dimethylates ‘Lys-20' of histone H4. Due to the functional importance of this gene and its known association with breast cancer^26^,^27^, we followed this gene mutation and performed high-depth (sequencing depth >10,000 reads) targeted mutational screening from samples at the MDS stage (TP1 and TP2). Unlike del(7q), this mutation was not detected in any of the previous MDS samples (Fig. 6f).

**Figure 7:**
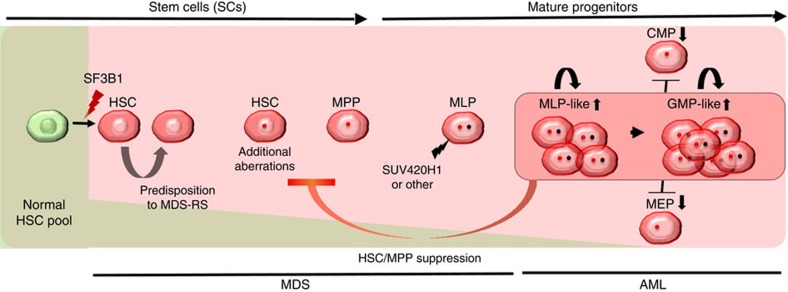
Predicted model for sequential acquisition of genetic lesions in MDS to AML transformation. *SF3B1* mutations are acquired in CD34^+^CD38^−^CD90^+^CD45RA^−^CD49f^+^ HSCs. As HSCs replenish downstream progenitors, mutations acquired in stem cells are also propagated to mature progenitors but without conferring self-renewal potential. However, as the mature progenitors such as MLPs harbouring a mutant *SF3B1* acquires a more ‘potent hit' such as *SUV420H1* mutation, they confer self-renewal potential and suppress the HSC to MPP transition.

Furthermore, mutational analysis of the HSC, MLP and GMP compartments (AML stage) revealed that *SF3B1* and *DNMT3A* mutations were present in all the compartments. However, *SUV420H1* mutation was not detected in HSCs but originated from MLPs and propagated to GMPs, supporting the hypothesis of mutations acquired at a MDS progenitor stage conferring self-renewal ability (Fig. 6g; Supplementary Fig. 8C). Taken together, our data suggest that *SF3B1* mutation precedes del(7q) aberration and is maintained throughout the disease evolution (Fig. 6h). Furthermore, acquisition of additional ‘potent' mutations such as *SUV420H1* in the ‘SF3B1-del(7q)' subclone in the MLP compartment led to the expansion of this MLP clone and hence transformation to AML (Fig. 7). Overall, our results provide evidence for the existence of MDS-ICs in the MDS-RS disease, which persist during the AML transformation and could potentially provide a reservoir of the pre-leukaemic stem cell, as well as play an important role during disease relapse.

## Discussion

Next-generation sequencing technologies have greatly improved our understanding of the genetic landscape in cancer. Previous work in AML and MDS demonstrated the existence of clonal heterogeneity that evolves upon disease progression and/or relapse^14^,^28^,^29^,^30^,^31^. To date, various studies including ours have shown that *SF3B1* is the most frequently mutated gene in MDS with a remarkably strong association with a specific disease phenotype, that is, the presence of ring sideroblasts^3^,^4^,^5^, suggesting that this mutation is casually linked to event-defining ring sideroblastic anaemia. However, it is noteworthy that studies so far have not determined the origins of *SF3B1* mutations within the haematopoietic compartment and the composition of the *SF3B1* subclones. Similarly, the functional heterogeneity of genetically defined *SF3B1* mutant subclones has not been delineated. *SF3B1* mutations are detected in low-risk disease and therefore, these patients in general have low frequency of other gene mutations^4^,^8^,^20^. However, the recurrent gene mutations that coexist with *SF3B1*-mutated patients are *TET2*, *DNMT3A* and *JAK2*. In fact, based on previous data, *TET2* (26%) and *DNMT3A* (21%) occur roughly at the same frequency in the *SF3B1* mutant patients, whereas mutations such as *ASXL1* coexist only in 8% of the *SF3B1*-mutated patients (Supplementary Table 3). Here we used WES, capture-based targeted high-depth sequencing, long-term culture assay, xenotransplantation as well as single-cell clonogenic assays to study the characteristics of *SF3B1* mutant haematopoietic cells. Mutational analysis of the *SF3B1* gene in haematopoietic stem/progenitor cells reveals that *SF3B1* mutations arising in phenotypically defined CD34^+^CD38^−^CD45RA^−^CD90^+^CD49f^+^ stem cells therefore unveil the MDS-ICs existence in RARS that propagate mutations to their myeloid progenitors, such as MPPs, GMPs and MEPs. This was also reflected in clonogenic assays where *SF3B1* mutations were detected either alone or in combination with other mutations in individual CFC colonies, therefore implying that *SF3B1* aberrations precede other gene mutations. Similar results have been described recently, in MDS patients with concurrent *SF3B1* and del(5q) aberrations, where *SF3B1* mutations were shown to be present in CD34^+^CD38^−^CD90^+^ stem cells in these patients^15^.

In our study, xenotransplantation of CD34^+^ BM from MDS samples in immunodeficient mice demonstrated the persistent long-term myeloid-restricted engraftment in the majority of cases. In contrast, NSG data from one congenital sideroblastic anaemia patient carrying an *ALAS2* mutation and other healthy donors showed a multi-lineage engraftment with high a percentage of the lymphoid/myeloid cell ratio. It is noteworthy that all four patients in our study had only one common genomic aberration, that is, *SF3B1*. In addition, 2/4 patients (MDS2 and MDS3) had a lower mutation rate (2–4 mutations per exome), which is less as compared with that in AML (≈13), chronic lymphocytic leukaemia (≈12), and was significantly lower as compared with multiple myeloma (≈33), hence representing a much more homogenous clonal population.

Notably, the genotypic analysis of pre- and post-transplanted cells demonstrate that in most of the cases *SF3B1* mutations were maintained; however, in one case (MDS3), *SF3B1* mutant clone increased from 27% in primary CD34^+^ cells to 47% in HEC samples. This suggests the presence of a mixed stem cell population (that is, *SF3B1* mutant/wild type), which when injected into immunodeficient mice resulted in the expansion of *SF3B1* mutant clones preferentially. Exclusive myeloid engraftment and the maintenance/increase in the *SF3B1* clone(s) in NSG xenografts suggest that a malignant HSC clone with *SF3B1* mutations either alone or in conjunction with other ‘cooperating factors' has a growth advantage or a negative feedback over the cohabitating normal HSC.

Interestingly, in all mice that have detectable human engrafted cells, we could see a similar subclonal architecture compared with MDS cells injected. Nevertheless, in some cases, the engrafting *SF3B1* daughter subclones represented only a small fraction of the injected cells (<5%), implying that some *SF3B1* subclones have a cell-intrinsic advantage (due to better engraftment ability, enhanced proliferation potential and/or additional factors) following xenotransplantation. Malignant clones with ‘isolated *SF3B1'* mutations are not always driven away by more ‘potent' mutant subclones (such as *DNMT3A* and/or *JAK2*) in NSG mice as was reflected by its MAB, which remained stable even though other subclones were fluctuating. Perhaps additional driver mutations when acquired by the *SF3B1* clones confer a subtle advantage to the new daughter subclone representative of early stages in disease progression, as evidenced in MDS1 (time point 1 versus time point 2). However, when these clones acquire more aggressive mutation(s), as is the case here for MDS2, a more ‘aggressive' mature progenitor clone with self-renewing capacity is generated, enabling the disease to evolve into AML. It is tempting to speculate that *SUV420H1* mutations confer a growth advantage based on the evidence that Suv420h (referred to both Suv420h1 and Suv420h2)-deficient MEFs and ES cells have been reported to have telomere elongation and epigenetic defects^32^. Further work needs to be done to study the role of *SUV420H1* mutation(s) in haematological diseases.

It is noteworthy that a small subclone with *SF3B1* mutation harbouring del(7q36) becomes a predominant clone in the sequential AML sample (MDS2), but with the additional acquisition of del(7q22). Therefore, *SF3B1* mutation in isolation might only have a minor role in disease transformation or progression to AML. However, similar to del(5q) aberrations^15^, it may instead be a strong driver mutation in MDS-RS and acquires additional mutations during the course of the disease progression.

Previous studies of functional heterogeneity in human leukaemias have used NSG mice to characterize leukaemia stem cells to decipher their engraftment potential, self-renewal capacity^33^,^34^,^35^,^36^. Nevertheless, Tim Ley's group was the first to report functional heterogeneity of genetically defined subclones in AML^14^. Their data suggested that xenotransplantation results in a marked decrease in the subclonal complexity of the AML samples and were not able to reliably predict disease relapse. However, the combination of our WES in xenotransplanted NSG mice and serial patient sampling enabled us to compare the clonal evolution observed in mice with that of patient BM compartment in MDS1 and MDS2. The changes in the spectrum of *SF3B1* mutant clones observed in the NSG mice were largely reflected in the patient's sequential BM cells where similar clonal changes were detected, although the *SF3B1* clonal fluctuation observed in NSG mice for MDS1 was more remarkable as compared with MDS2.

This study demonstrates that *SF3B1* mutations can originate in rare CD34^+^CD38^−^CD90^+^CD49f^+^ HSCs, and MDS-ICs are restricted to these rare HSC fractions in our cohort of MDS patients with ring sideroblasts. Although our data are limited to five patients, the presence of *SF3B1* mutations in CD34^+^CD38^−^CD90^+^CD45RA^−^CD49f^+^ HSCs provides new insight into the MDS stem cells. Furthermore, our data demonstrate that *SF3B1* mutations could act as an initiating event in MDS-RS disease, as has been suggested by previous studies^3^,^5^. We could not, nevertheless, exclude that in other RARS cases, mutations such as TET2 and ASXL1 (not studied here) could also act as initiating mutations. Further analysis of a larger cohort of patients, using single-cell or the clonogenic assay should address this point.

MDS2 patient progression to AML illustrates the concept of a switch from ‘HSC disease' to a ‘progenitor disease'. Here, we show that in some cases MDS-RS can originate from the stem cells and at a later stage of the disease *de novo* mutation(s) acquired by the progenitor cells can confer the self-renewal capability to the MDS progenitor cells that drives leukaemic transformation. This novel observation confirming the presence of MDS-IC at the AML stage provides new insight into the MDS/AML hierarchy. Our results also suggest that MDS-IC at the AML stage can constitute a pool of pre-leukaemic cells, providing a reservoir of cells that could be responsible for relapse. Although mutations such as *SF3B1* are not driving mutations at the AML stage, they provide a genuine target for curative therapies and give the opportunity to therapeutically target at the same time the AML blasts as well as pre-leukaemic cells.

## Methods

### Patients

WES data were available for 12 MDS-RS patients (Supplementary Table 1) and one congenital sideroblastic anaemia patient. In addition, one MDS-RS patient with *SF3B1* mutation was also included in this study. All patient samples (*n*=14) were received from the King's College London Haemato-Oncology Tissue Bank. All patients had provided written informed consent for the samples to be used for all the experiments in accordance to the King's College London Haemato-Oncology Tissue Bank research ethics protocol (08/H0906/94). Demographic and clinical characteristics of the studied patients are detailed in Supplementary Table 1. All patients were risk stratified according to IPSS categories. The clinical variables for all patients were ascertained at the time of sample collection. Sequential (post AML transformation) samples from MDS2 was also available (Supplementary Tables 1 and 4).

BM aspirates from 6/12 MDS-RS (MDS1, MDS2, MDS3, MDS4, MDS6 and MDS14) all containing the *SF3B1* mutations (K700E, *n*=2; H662Q, *n*=3) and one congenital sideroblastic anaemia patient (MDS5, wild-type *SF3B1*) were selected for *in vivo*, *in vitro* experiments and/or haematopoietic cell separation (Supplementary Tables 1 and 4).

### Xenotransplant assays

MNCs were isolated from the BM cells by centrifugation using Ficoll-Paque PLUS (GE Healthcare Life Sciences, Buckinghamshire, UK). The cells were processed within 24 h following collection using an Easysep Human CD34-positive selection kit and Easysep magnet (StemCell Technologies, Vancouver, Canada) according to the manufacturer's instructions to enrich CD34^+^ cells from patient samples. Mesenchymal stem cells were isolated from the CD34^−^ fraction during the CD34^+^ cell selection. Cells were seeded at a concentration of 1.10^6^/cm^2^, in DMEM low glucose (Life Technologies, Paisley, UK) supplemented with fetal bovine serum (Mesenchymal Stem Cell Qualified, Life Technologies). Culture media was replaced 3 days later, and cells were frozen at passage-2 maximum.

NSG mice were a kind gift of Dr Leonard Shultz (The Jackson Laboratory). All animal experiments were performed in accordance to Home Office and CRUK guidelines. Female NSG mice used in this study were 8 to 12 weeks old at the start of the experiments. Before transplantation, mice received a sub-lethal dose of radiation (375 cGy) from a caesium-137 source. Direct intra-BM injection was performed in the tibia with 0.65 × 10^5^ to 2 × 10^5^ BM CD34^+^ cells from patients and healthy donors.

Engraftment was assessed over time by intra-tibia aspiration under isoflurane anaesthesia, and the BM was immunophenotyped by the presence of mCD45^−^, hCD45^+^, hCD33^+^, hCD19^−^ and hCD3^−^ (using anti-mouse and anti-human antibodies from BD Biosciences, Oxford, UK) cell populations. After 18–20 weeks, mice experiments were terminated and cells were harvested from pooled BM (femurs, tibias, pelvis and/or spine). Live cells were stained and sorted on the hCD45^+^ phenotype using FACS Aria SORP (BD Biosciences). Sorted cells were washed in PBS and harvested to later perform genomic analysis.

### CD45 Immunohistochemistry

Femurs were collected from engrafted and non-engrafted mice, washed in PBS, fixed overnight at 4 °C in 4% paraformaldehyde (methanol free). Samples were washed in PBS once before being washed in water. Bones were incubated 2 h on ice in 8% HCl solution for decalcification. Samples were rinsed with water and stored in PBS before immunostaining.

Tissues were fixed in 10% neutral buffered formalin overnight and processed for paraffin embedding and sectioned at 4 M. Sections were microwaved for 15 min in 0.01 M sodium citrate buffer pH 6 for antigen retrieval. Sections were blocked with 1.6% H_2_O_2_/PBS and then 10% normal horse serum/1% BSA and stained for 1 h with mouse anti-CD45 (Dako M0701, Cambridgeshire, UK) at 1/200 dilution. Secondary antibody was used with biotinylated horse anti-mouse IgG (Vector Labs, Peterborough, UK), followed by ABC reagent (Vector Labs), developed with DAB (Vector Labs) and counterstained with haematoxylin. Pictures were taken using an inverted microscope and analysed with NIS-Elements software from Nikon.

### CD45 immunohistochemistry

Femurs were collected from engrafted and non-engrafted mice, washed in PBS, fixed overnight at 4 °C in 4% paraformaldehyde (methanol free). Samples were washed in PBS once before being washed in water. Bones were incubated 2 h on ice in 8% HCl solution for decalcification. Samples were rinsed with water and stored in PBS before immunostaining.

Tissues were fixed in 10% neutral buffered formalin overnight and processed for paraffin embedding and sectioned at 4 M. Sections were microwaved for 15 min in 0.01 M sodium citrate buffer pH 6 for antigen retrieval. Sections were blocked with 1.6% H_2_O_2_/PBS and then 10% normal horse serum/1% BSA and stained for 1 h with mouse anti-CD45 (Dako M0701) at 1/200 dilution. Secondary antibody was used with biotinylated horse anti-mouse IgG (Vector Labs), followed by ABC reagent (Vector Labs), developed with DAB (Vector Labs) and counterstained with haematoxylin. Pictures were taken using an inverted microscope and analysed with NIS-Elements software from Nikon.

### Isolation of HSCs and progenitors

BM MNCs were separated from total BM cells by density-gradient sedimentation with Histopaque-1077 (Sigma-Aldrich Co. LLC). Up to 1 × 10^8^ MNCs were resuspended in 350 μl of auto-MACS running buffer and subsequently used for immunomagnetic enrichment of CD34^+^ cells using MACS. CD34^+^ cells were enriched by positive selection as per the manufacturer's instructions (Miltenyi Biotec Ltd, CD34 MicroBead Kit Human). CD34^+^ cells were kept on ice for subsequent use and CD34^−^ cells were stored as viable cells in Synth-A-Freeze media (Life Technologies) at −80 °C.

Up to 1 × 10^6^ CD34^+^ cells were stained with the following antibodies: 5 μl of CD45 (APC-eFluor 780, clone HI30), 1 μl of CD49f (FITC, clone GoH3), 5 μl of CD34 (PE-Cy7, clone 4H11), 5 μl of CD38 (APC, clone HIT2), 5 μl CD135 (PE, clone BV10A4H2) (eBioscience), 5 μl of CD45RA (BV 510,clone HI100), 2 μl of CD90 (Alexa Fluor 700, clone5E10) (Biolegend UK Ltd) and a lineage-specific antibody cocktail (1 μl of CD2 biotin, 1 μl of CD3 biotin, 1 μl of CD14 biotin, 1 μl of CD16 biotin, 1 μl of CD19 biotin, 1 μl of CD24 biotin, 1 μl of CD56 biotin, 1 μl of CD235a biotin; together with 5 μl of Streptavidin eFluor 450, all by eBiosciences and CD66b (V450) (BD Biosciences). Propidium iodide was added to the cell suspension before sorting to exclude dead cells. Cells were sorted on a BD FACS Aria SORP (San Jose, CA) operating in a four-way purity sort mode, and collected into 1.5-ml microfuge tubes. Our cell populations of interest were defined by hierarchical gating, which was largely similar to the one used by Notta *et al.*^23^; HSCs as Lin^−^CD45^+^CD34^+^CD38^−^CD45RA^−^CD90^+^CD49f^+^, MPPs as Lin^−^CD45^+^CD34^+^CD38^−^CD45RA^−^CD90^−^CD49f^−^, GMPs as Lin^−^CD45^+^CD34^+^CD38^+^CD135^+^CD45RA^+^ and MEPs as Lin^−^CD45^+^CD34^+^CD38^+^CD135^−^CD45RA^−^ (Supplementary Fig. 1). Moreover, in the case of the AML sample, CD123 antibody (PE, clone: 6H6, eBioscience) was used instead of CD135 to define the CMP, MEP and GMP compartments.

After sorting, the purity of MPPs, GMPs and MEPs were checked and a sorting purity of ≥97% was routinely obtained. Cells were spun down after sorting, supernatant was discarded and cells were either stored at −80 °C and/or resuspended in Karnoy's solution to later perform FISH analysis.

### Whole-genome amplification

WGA of cells was performed with the GenomePlex Single Cell Whole Genome Amplification kit (WGA4, Sigma-Aldrich Co, LLC) and/or GenomePlex SeqPlex XE kit (SEQXE, Sigma-Aldrich Co., LLC). WGA amplification was performed according to the instructions of the manufacturer, along with a no-cell reaction as a negative control and a reaction of human tissue genomic DNA (gDNA) as a positive control.

For patient samples where only one mouse was used in *in vivo* experiments, two independent amplification experiments were performed using two different amplification kits (that is, WGA4 and SEQXE). In addition, all patients samples, the HSCs and MPPs were also amplified by two different amplification kits (WGA4 and SEQXE), while the GMPs and MEPs were amplified by using the SEQXE WGA kit.

### WES and data analysis

Thirteen MDS-RS patients were selected for WES, using DNA from CD34^+^ cells (primary BM sample) in all cases and paired constitutional DNA from 11 cases (skin, *n*=7; CD3^+^ *n*=4). One additional MDS-RS patient was subjected to targeted mutational analysis. The WES data have been deposited in the NCBI Sequence Read Archive under accession code XXX.

CD45^+^CD33^+^ cells derived from mice (human engrafted cells) from four of these patients (MDS1, MDS2, MDS3 and MDS4) were also subjected to WES. WES was also performed on three LTC-derived samples from three patients (for more details, see Supplementary Table 4). For cases where WGA was performed using two independent WGA kits, both WGA samples were independently processed for WES.

gDNA (non-WGA, 100–500 ng) or WGA DNA (500 ng) was processed for exome sequencing (Agilent V4) and sequenced on the Illumina HiSeq2000 (Paired end V3 chemistry) according to manufacturer's instructions. Base calling was generated by the Illumina RTA software. Demultiplexing and conversion of basecalls to fastq was performed by Casava version 1.8.2, filtering out poor-quality reads fastq files concatenated. Alignment, realignment and recalibration was performed using Burrows–Wheelers aligner^37^ and GATK^38^, respectively, according to the Broad Institute best practices for individual samples. VarScan 1.3.4 (refs 39, 40) was subsequently used to call somatic variants on pileup files (Samtools)^41^ for paired tumour and normal (skin or CD3^+^ T cells) tissue. Reads with an alignment score of <10, base quality score <15 and fold strand bias >10 were excluded. Pindel^42^ was also used to screen for more computationally challenging Indel mutations at specific loci, such as for FLT3 ITD. All resulting variants were passed through ANNOVAR^43^ using refseq annotation, and variants deemed to cause protein changes and not found in dbSNP135, esp5400 and 6500, and 1,000 genomes databases at >0.001 population allele frequency were passed. Genomic duplicated regions were also filtered out unless associated with known mutations. Somatic mutations were subsequently passed when the somatic *P* value (VarScan, Fisher's exact *T*-test) was <0.01, had ≥3 reads supporting mutation, were present in paired normal skin tissue at <20% (or for paired *t*-cells <50%) at <20% of the paired tumour tissue and had an allele burden of >5%. For isolated HEC CD45^+^CD33^+^ cells, candidate mutations were only passed if they were present in >1 HEC sample or found concurrently in one or more HEC experiment and LTC-derived sample or primary CD34^+^ cells for the same patient. Where only WGA-derived data from HEC were available for one engraftment experiment, or a combination of WGA and non-WGA data from more than one engraftment experiment, mutations were only passed when they were found concurrently in either: (1) WGA HEC and non-WGA HEC-independent experiments; (2) repeat WGA DNA samples from the same engraftment experiments or; (3) WGA HEC experiments and LTC-derived sample, CD34^+^ or HEC experiments derived from non-WGA DNA. For WGA-derived HEC, a stand-alone analysis was also performed for individual experiments. For this, additional false-positive filters were applied to remove additional WGA-derived artefacts with positional and read-length distribution bias, as well as variant and mapping quality biases. Variants with homopolymer runs of five or more flanking the mutation were also filtered out. WGA-derived variants from control WGA experiments were also removed. For MDS3, only variants found in at least two of the three animal experiments from WGA-derived data were included in the final list due to increased artefactual events. Stand-alone analysis of non-WGA HEC cells were likewise performed but only using filters up to step 7 above.

### Mapping loss of heterozygozity across chromosomes by WES

Loss of heterozygosity was mapped from WES data according to the following criteria: variants were called as described above. Variants found in the 1,000 genome project database with a population allele frequency <0.001 were selected and filtered out for known genomic duplicated regions, read depth <20 and somatic status according to VarScan. The filtered set of variants was then plotted graphically for the required chromosome.

### Exome coverage

Exome coverage was calculated utilizing GATK Depth Of Coverage script against intervals that the Agilent V4 Sureselect kit capture probes were based on. For non-WGA sample, mean base coverage across the targeted region ranged from × 77 to 115 and for >98% of the exome base coverage was >10 reads in all cases, based on 9–13 gigabase of sequence data. For the same amount of sequence data on average, WGA-derived exome data yielded an average base coverage of × 55–75 and 65–83% (average 74%) of exome covered ≥10 reads.

### Sequencing validation experiments

All somatic mutations were confirmed independently by sequencing from three independent PCR reactions from all experiments (CD34^+^ day-0 samples, HEC from mice, LTC and CFC), which enabled us to quantify the MAB more accurately in all cases.

Primers for candidate gene variants were designed by using Primer 3 program^44^ with default settings. Using relevant candidate gene-specific primers and GoTaq Hot Start Colorless Master Mix (Promega), PCR amplification was performed on all primary CD34^+^ samples, HEC samples, LTC-derived cells and pooled CFC by following manufacturer's protocol. The acquired nature of the mutations was also confirmed by their absence in paired constitutional DNA. In the case of WGA-derived candidate mutations, validation was also confirmed in at least one gDNA (non-WGA) sample from that particular patient.

In the case of WGA-derived candidate mutations, validation was also confirmed in at least one gDNA (non-WGA) sample from that particular patient. The degree of quantitative accuracy of the allele burdens for WGA DNA was validated by performing WES on paired gDNA (non-WGA) and WGA DNA from the samples (Supplementary Fig. 3). For this, we observed a similar distribution of known single-nucleotide polymorphisms across the exome between the gDNA (non-WGA) and WGA DNA. To validate this further, we also performed mutational screening for 10 genes on paired gDNA (non-WGA) and WGA DNA from two (MDS1 and MDS2) independent patients (Supplementary Fig. 3). MAB for specific mutations was also shown to be comparable between gDNA (non-WGA) and WGA DNA in targeted amplicon sequencing in these control experiment.

For MDS1, all the mutations including *SF3B1* were confirmed in all mice experiments using gDNA (non-WGA) DNA. In addition, all mutations were also confirmed in LTC (non-WGA) and CFC (non-WGA) samples. For MDS2, all the mutations were confirmed in amplified DNA from mice; however, *SF3B1* and four of the additional mutations were confirmed on gDNA (non-WGA) from mice. In addition, all mutations were also confirmed in LTC (non-WGA) and CFC (non-WGA) samples. For MDS3, all the mutations including *SF3B1* identified in two animals from amplified DNA were also found in one gDNA (non-WGA) from mice. In addition, all mutations were also confirmed in LTC (non-WGA) and CFC (non-WGA) samples. For MDS4, *SF3B1* and one additional mutation was confirmed in mouse gDNA (non-WGA).

Furthermore, all somatic mutations were confirmed independently by sequencing from three independent PCR reactions from all mice experiments, which enabled us to quantify the MAB more accurately in all cases. The degree of quantitative accuracy of the allele burdens for WGA DNA was validated by performing WES on paired gDNA (non-WGA) and WGA DNA from the samples (Supplementary Fig. 2). For this, we observed a similar distribution of known single-nucleotide polymorphisms across the exome between the gDNA (non-WGA) and WGA DNA. To validate this further, we also performed mutational screening for 10 genes on paired gDNA (non-WGA) and WGA DNA from 2 (MDS1 and MDS2) independent patients (Supplementary Fig. 2). MAB for specific mutations was also shown to be comparable between gDNA (non-WGA) and WGA DNA in targeted amplicon sequencing in these control experiments.

Here, PCR amplicons were normalized, mixed in batches and converted into Illumina sequencing libraries utilizing transposon-based Illumina-Nextera technology (Illumina), designated nextera-amplicon libraries here. These libraries were sequenced on the Illumina MiSeq utilizing version 2 chemistry, 150–250 paired-end reads and dual indexes allowing multiplexing of 96 samples independent of sequencing experiments utilizing the nextera-amplicon methodology.

### Primers used for validation

Target genes oligo sequence (5′ to 3′)

SF3B1_14f CCAACTCATGACTGTCCTTTCTT

SF3B1_14r GGGCAACATAGTAAGACCCTGT

SF3B1_15f TTGGGGCATAGTTAAAACCTG

SF3B1_15r TTCAAGAAAGCAGCCAAACC

JAK2_E14f TCCTCATCTATAGTCATGCTGAAA

JAK2_E14r CTGACACCTAGCTGTGATCCTG

DNMT3A_E8r TCTTGCCTCATTCAGATGGA

DNMT3A_E8r CCTGGGATCAAGAACCTTCC

DNMT3A_E15f ACCAGGGCTGAGAGTCTCCT

DNMT3A_E15r AGGCTCCTAGACCCACACAC

FBLIM1_f CGAGAAGGGTTTGTGCACTG

FBLIM1_r TCCCAGGTTCACGCCATT

HIST1H2AC_f CGCGACAACAAGAAGACTCG

HIST1H2AC_r GCAGTGAGGTTAGCTCTTCC

HSPA12A_f CCAGTGTGCCCTCAAAAGTC

HSPA12A_r CTTATGCAGTTCGGGGACAC

RETNLB_f AAGAATGGGCAAGGGGTCTC

RETNLB_r ATGGGGAGGATAGCTCATGG

SMC6_f TGGAACATTAGGGCCAAATCT

SMC6_r GCCAAGTTTTGAGGACTCCA

SP3_f AGCCAGACTGACTTGTTCTTTG

SP3_r CTGGAGAACGCCCTTTTGTT

TH_f CACGGATGTGTAGCAAAACG

TH_r CCTGCTTTTGCTCCCTAAGA

NAGA_f CGTAGTCAGCCAGGAAAGGA

NAGA_r GCTCAGCAAGGTCATGGTTT

NMNAT2_f CTCTCTCACTCCTCCAGCTG

NMNAT2_r TGTGGAAGAATGGAAGCGGA

NR0B2_f GCAAAAGCATGTCCCCAAGA

NR0B2_r CAGTCTTGTCCTTTGGTGGC

SPATA13_f CGCCGTGTGTGAGATTCTC

SPATA13_r CAGCCTCAGTCACATTGCTC

TLL2_f CCCCTCAATACAGTCCCCAA

TLL2_r AGGCTCTATTGCTCCTGGTG

G3BP1_f AGTCAGTGCCATGATTTTACCA

G3BP1_r ACAATGCAAGTTCTTATGCAGC

ABCB11_f AACAGACCAGCACTCACCTT

ABCB11_r TCATAAAACAGAGCAACAACCAG

CCDC17_f CTTTCCAGAACTGCTCAGCG

CCDC17_r GAGACCCGACTAAGCGGATC

ME1_f TACCTTGGCTTCCGAAACAC

ME1_r ACAGGCATGAACCACTGTGC

CAMTA1_f GTTCTCTGCAGGGAGGAGTG

CAMTA1_r CTGCCCTAAATCGTGCTCTC

PTPDC1_f CGCAAATTAGAGGATCACAACA

PTPDC1_r CCCTTCCTCCAGAAATGTGT

MYH14_f AAGACCCGGATATGGGAAGT

MYH14_r TCCTCCCTCAACAGATCCAC

### Colony-forming cell

Five hundred cells from the BM CD34^+^-enriched fraction were plated in triplicate in 0.5 ml of MethoCult H4434 (StemCell Technologies). At day 14 of culture, the numbers of colonies were scored. Single-cell colony images were acquired with a Zeiss AxioVert 40 CFL microscope using × 5 lens and connected to Canon PowerShot A640 digital camera. Cells were picked, harvested and washed twice with PBS. All CFC colonies were screened for *SF3B1* and additional patient-specific mutations.

### LTC assay

LTC assays were performed by plating 10^3^ CD34^+^ BM cells in quadruplets on irradiated MS-5 murine stromal cells and cultured in Myelocult H5100 (StemCell Technologies) in the presence of cytokines (20 ng ml^−1^ G-CSF, 20 ng ml^−1^ IL-3 and 20 ng ml^−1^ TPO from PeproTech, London, UK). After 5 weeks, live cells were stained and sorted on the human CD45^+^ phenotype using the FACS Aria SORP (BD Biosciences). Sorted cells were washed in PBS, harvested and stored as cell pellet and/or resuspended in Karnoy's solution to later perform FISH and sequencing analysis.

### Genotyping of single-cell clones and/or colonies

CFU-GM and BFU-E colonies were harvested for four patients (MDS1, MDS2, MDS3 and MDS4) at day 14, and washed twice with PBS. All colonies were subjected to WGA (single-cell WGA, Sigma-Aldrich). All CFC colonies were screened for *SF3B1* and additional patient-specific point mutations. Amplicon libraries were amplified through PCR using the primers specific to gene mutations and subjected to Nextra-Illumina sequencing. Gene mutation for each single colony was called when >30% of the sequencing reads were carrying a mutant allele.

### Amplicon sequencing for cell fractions

WGA-derived DNA from cell fractions (HSCs, MPPs, GMPs and MEPs) were subjected to sequencing. Candidate gene mutations from the WES data were followed-up specifically. These mutations were also screened in mesenchymal stem cells (*n*=4) derived from the concurrent patients. Furthermore, all somatic mutations were confirmed independently by sequencing from ≥2 independent PCR reactions. Sequencing was performed using Nextra Technology (Illumina) and MiSeq platform (Illumina) as described previously.

MAB is presented as an average of at least two independent Illumina-Nextera validation experiments combined with data from exome sequencing where available. In this way, the variation expected from sampling error when working with small progenitor cell populations was minimized. The average sequencing coverage across all amplicons was ≥800 × . This coverage enabled us to reliably detect mutant clones down to ≥1% MAB, defined as the proportion of sequence reads containing the mutation.

*SF3B1* gene mutation were analysed in haematopoietic cell fractions (HSCs, MPPs, GMPs and MEPs) by PCRs directly on 30–50% of the cell fraction with subsequent sequencing. For one additional patient (MDS6), which was not used for *in vivo* and *in vitro* experiments, *SF3B1* mutation status was assessed in cell fractions in the same manner.

### Fluorescence *in situ* hybridization

Cells were pelleted, washed twice in PBS and then resuspended in 0.5 to 1 ml of Karnoy's solution (3:1 methanol/acetic acid glacial, purchased from Sigma-Aldrich and Fisher Chemical, respectively) and then stored at 4 °C before hybridization. For interphase FISH, fixed cells were centrifuged and an appropriate amount of supernatant was removed. Pelleted cells were then transferred onto the slides and hybridized with a XL 7q22/7q36 (Metasystems) locus-specific probe, which detects deletions in the long arm of chromosome 7 (Supplementary Fig. 6). The orange-labelled probe targets a specific region at 7q22 and the green-labelled probe binds specifically to 7q36. In addition, a blue (aqua)-labelled probe, which hybridizes to the centromere of chromosome 7 functions as a reference probe. Fluorescence images were obtained with the help of fluorescence microscopy. In all, 200–600 interphase cells were analysed per sample.

### Statistical analysis

Prism Version 6 software (GraphPad) was used for statistical analysis. Data are presented as the mean±s.e.m. Statistical analysis was performed using the two-tailed Student's *t*-test for comparison of two groups to determine the level of significance.

## Additional information

**Accession codes**: Data available on EBML Nucleotide Sequence Database (http://www.ebi.ac.uk/ena) with the accession number: PRJEB12004.

**How to cite this article:** Mian, S. A. *et al.* SF3B1 mutant MDS-initiating cells may arise from the haematopoietic stem cell compartment. *Nat. Commun.* 6:10004 doi: 10.1038/ncomms10004 (2015).

## Supplementary Material

Supplementary Information Supplementary Figures 1-8, Supplementary Tables 1-5, Supplementary Note 1 and Supplementary References

## Figures and Tables

**Table 1:** Engraftment of sorted haematopoietic cells in NSG.

	***SF3B1*** **mutation CD34**^**+**^ **cells**	**Mouse model**	**Cells injected**	**Engrafted mice**	**hCD33% cells hCD19% cells (12 weeks)**	***SF3B1*****MAB in CD34**^**+**^ **cells (day 0)**	***SF3B1*****MAB in HEC (12 weeks)**
Lin^−^CD45^+^CD34^+^CD38^−^							
							
HSC CD45RA^−^CD90^+^CD49f^+^	H662Q	NSG	1,629	1/1	100%0.00%	46%	46%
MPP CD45RA^−^CD90^−^CD49f^−^	H662Q	NSG	29,944	0/1	NA	NA	NA
							
Lin^−^CD45^+^CD34^+^CD38							
							
MLP CD45RA^+^CD90^−^	H662Q	NSG	51	0/1	NA	NA	NA
CMP CD45RA^−^CD135^+^	H662Q	NSG	16,231	0/1	NA	NA	NA
GMP CD45RA^+^CD135^+^	H662Q	NSG	28,217	0/1	NA	NA	NA

FACS, fluorescence-activated cell sorting; GMP, granulocyte macrophage progenitor; HEC, human engrafted CD45^+^CD33^+^ cell; HSC, haematopoietic stem cell; MAB, mutant allele burden; MDS, myelodysplastic syndrome; MDS-IC, MDS-initiating cell; MDS-RS, MDS patients with ring sideroblast; MPP, multipotent progenitor; NA, not applicable; NSG, NOD/SCID/IL2rγ^−OD^ mice.

The table shows the engraftment of FACS-isolated haematopoietic cell fractions injected into NSG mice. Progenitor cells from one MDS-RS patient were isolated and injected separately into NSG mice. After 12 weeks, mice were killed and the bone marrow was analysed. Only the rare HSC fraction gave rise to a myeloid-restricted engraftment, indicating the nature of the MDS-ICs in this rare HSC fraction.
