# Supplementary material for: SF3B1 mutant MDS-initiating cells may arise from the haematopoietic stem cell compartment
Source: Nat Commun. 2015 Dec 8;6:10004. doi: 10.1038/ncomms10004 (PMC4686651; doi:10.1038/ncomms10004)
Supplement: Supplementary Information — Supplementary Figures 1-8, Supplementary Tables 1-5, Supplementary Note 1 and Supplementary References [file ncomms10004-s1.pdf]

Supplementary Figure 1: WES data showing the clonality of the MDS-RS patients.

Prediction of order of genetic lesions in MDS-RS patients with one or more candidate driver mutation as identified by whole-exome sequencing (followed by confirmation by target mutational sequencing). This calculation was performed based on the MAB of the mutated genes. Vertical lines represent MDS-RS patients. SF3B1 and known myeloid related gene mutations are shown using labelled coloured circles. Other coloured circles represent patient specific mutations detected through whole-exome sequencing (see supplementary table no 2 for more details).

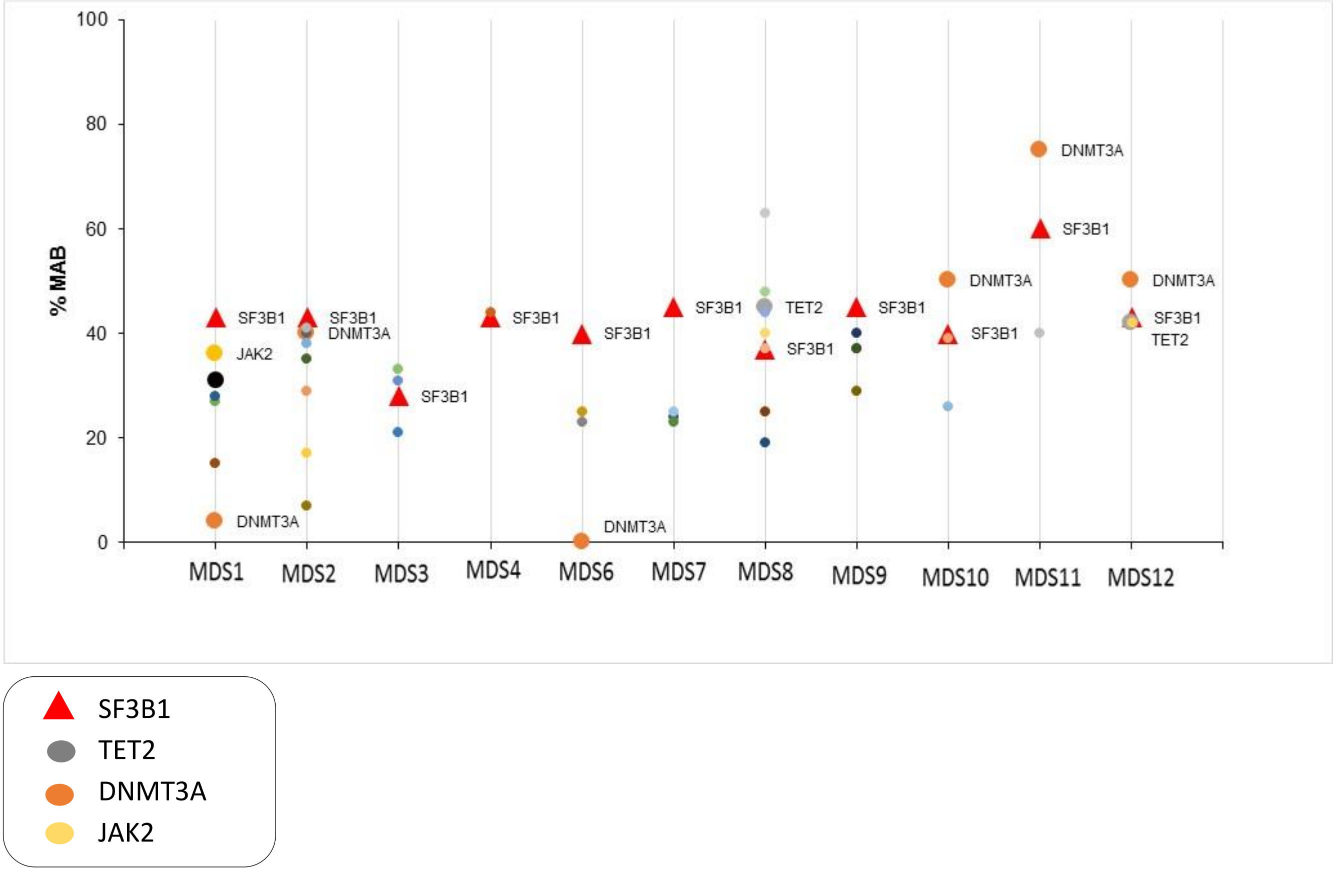

Supplementary Figure 2: Flow cytometry plots showing the gating strategy.

(A) Representation of the isolated haematopoietic stem cell subpopulations. Viable lineage negative $CD34^+$ cells were defined by hierarchical gating on a combination of forward (FSC) and side scatter (SSC), propidium iodide (not shown), CD45 and a lineage cocktail. These $CD34^+$ cells were further gated first into $CD34^+CD38^-$ and $CD34^+CD38^+$ populations and then subdivided into either HSCs ($CD34^+CD38^-CD90^+CD45RA^-CD49f^+$), MPPs ($CD34^+CD38^-CD90^-CD45RA^-CD49f^-$) or into GMPs ($CD34^+CD38^+CD135^+$ $CD45RA^+$) and MEPs ($CD34^+CD38^+CD135^-CD45RA^-$). (B) FACS profile of bone marrow progenitors cells for MDS-RS patients.

A

Healthy donor

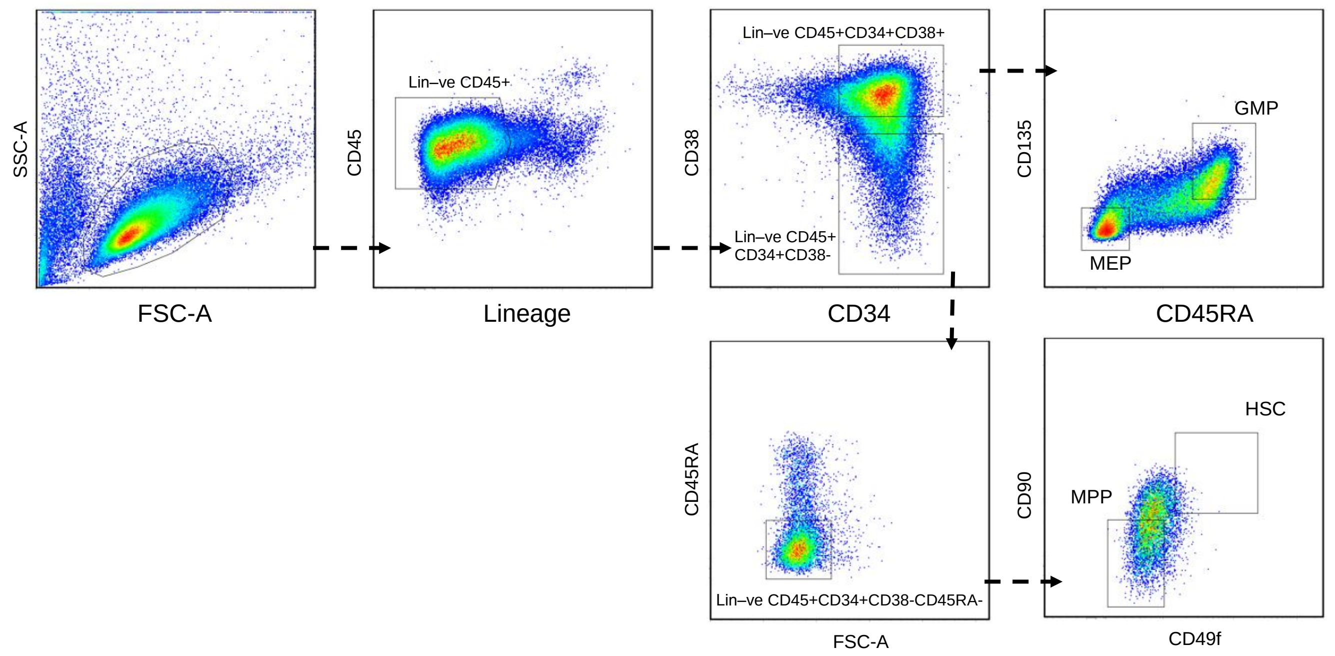

B

MDS1

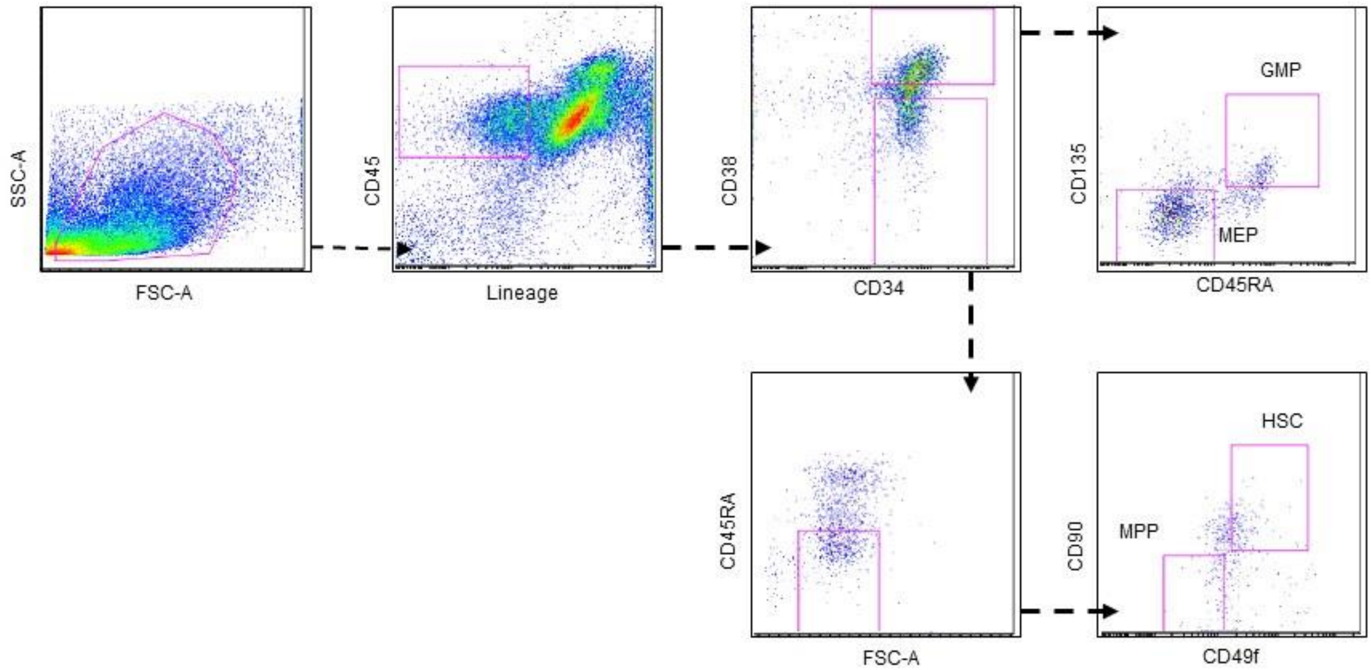
SSC-A
FSC-A
CD45
Lineage
CD38
CD34
CD135
CD45RA
GMP
MEP
CD45RA
FSC-A
CD90
CD49f
HSC
MPP

MDS2

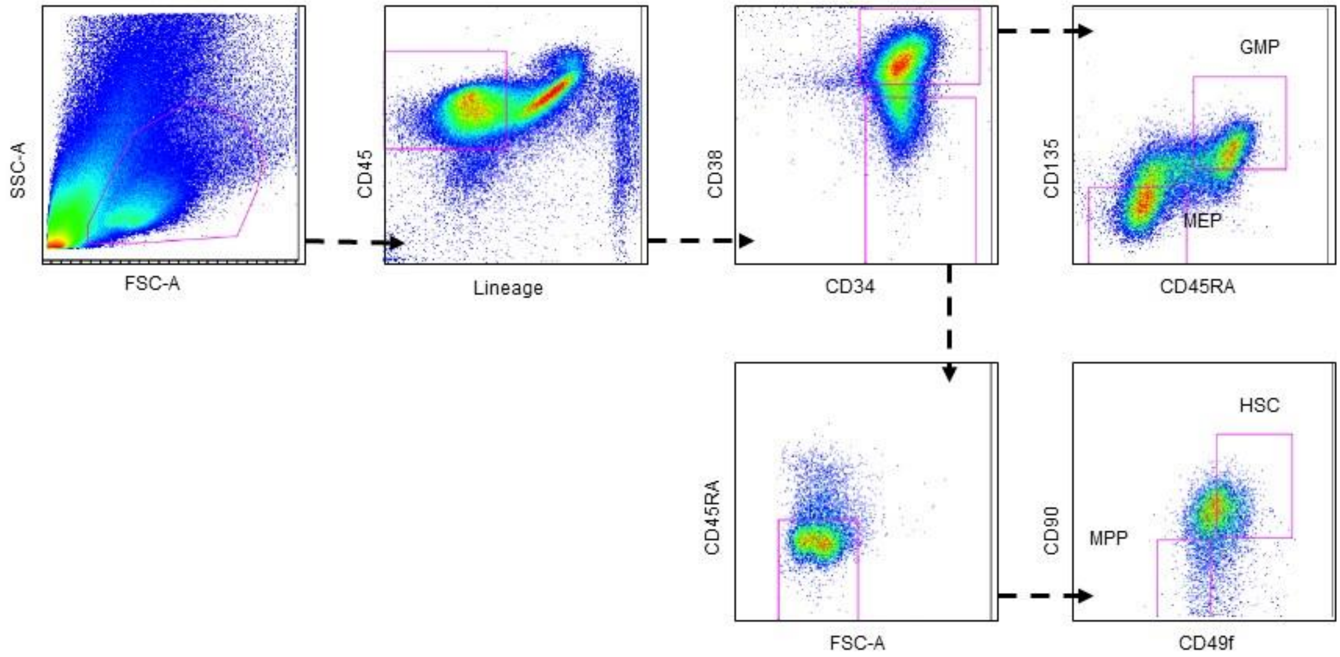
SSC-A
FSC-A
CD45
Lineage
CD38
CD34
CD135
CD45RA
GMP
MEP
CD45RA
FSC-A
CD90
CD49f
HSC
MPP

MDS4

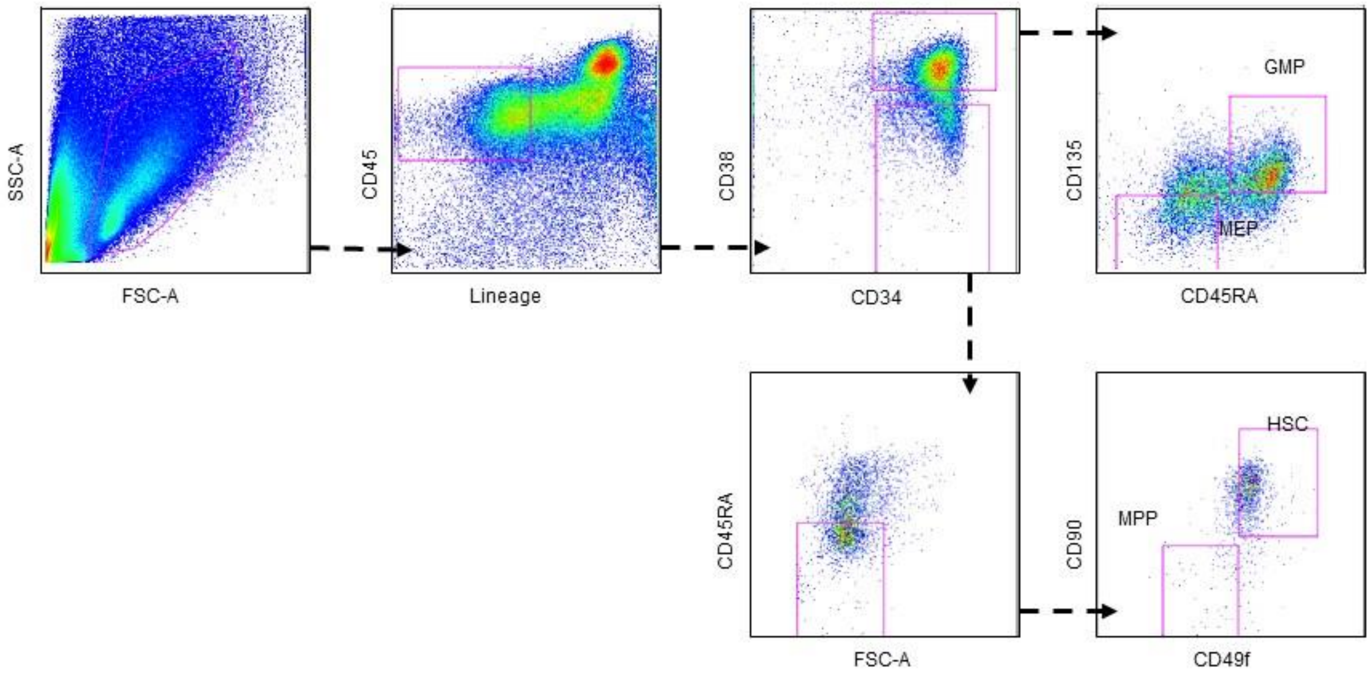
SSC-A
FSC-A
CD45
Lineage
CD38
CD34
GMP
CD135
MEP
CD45RA
CD45RA
FSC-A
HSC
MPP
CD90
CD49f

MDS6

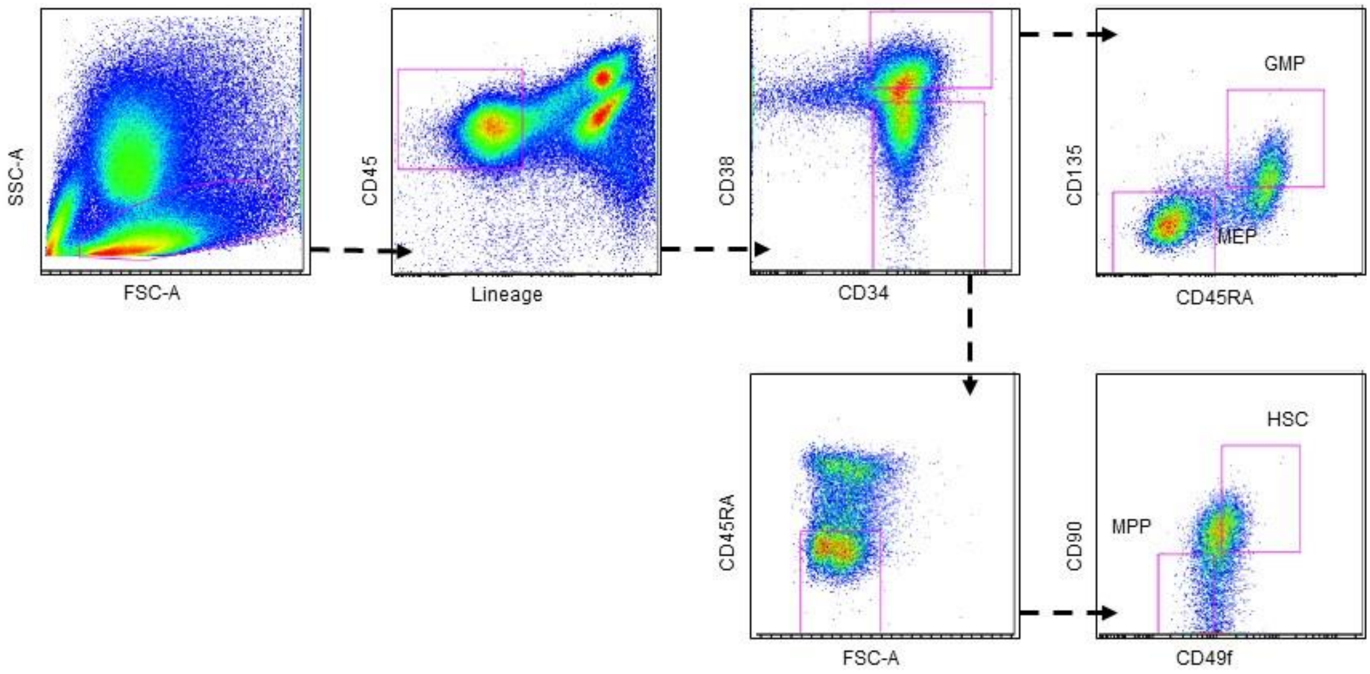
SSC-A
FSC-A
CD45
Lineage
CD38
CD34
GMP
CD135
MEP
CD45RA
CD45RA
FSC-A
HSC
MPP
CD90
CD49f

Supplementary Figure 3: Validation of gDNA vers WGA-DNA through SNV profiling by whole-exome sequencing and targeted-mutational analysis.

(A) Comparison of SNV profiles from control exome experiments based on gDNA vs WGA-DNA. Paired exome experiments (1-4) of gDNA (non-amplified) vs WGA-DNA were processed and analysed in VarScan as detailed in experimental procedures. Variants were selected and plotted based on being called germline or LOH in VarScan, due to presence at similar levels in both samples of a pair or significant allele burden (AB) change between the two respectively, had >20 reads depth and had >10% AB in unamplified experiment data. Chromosome 3 data is exampled here as representative figure. Panel 1 and panel 2 are based on the experiment of human $CD33^+$ cells isolated post mouse engraftment. Panel 3 is based on primary patient $CD34^+$ cells. Panel 4 is based on patient skin material. Panel 1, panel 3 and panel 4 show all variations as detailed above. Panel 2 shows sub-fraction of panel 1 that is found in dbSNP137. (B) Comparison of somatic mutations between paired gDNA vs WGA-DNA. Mutant allele burden of 5 gene mutations in primary $CD34^+$ bone marrow and $CD33^+$ HEC cells from MDS1 and MDS 2. MAB- Mutant Allele Burden, gDNA- Genomic DNA, WGA-Whole-genome amplified DNA.

A

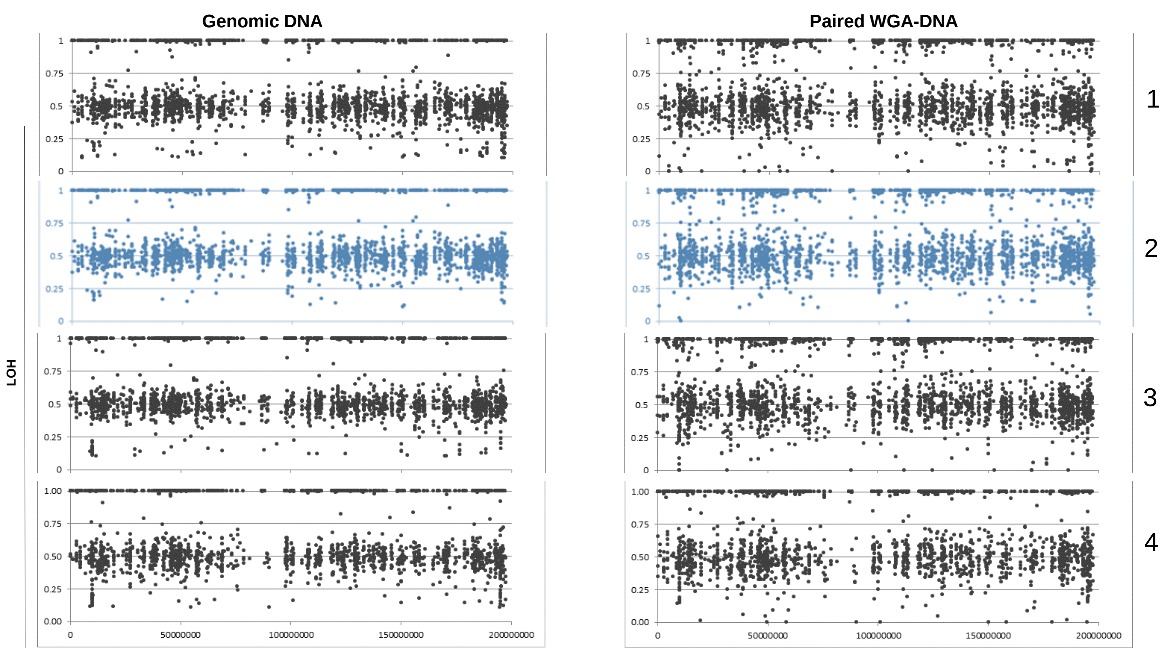

B

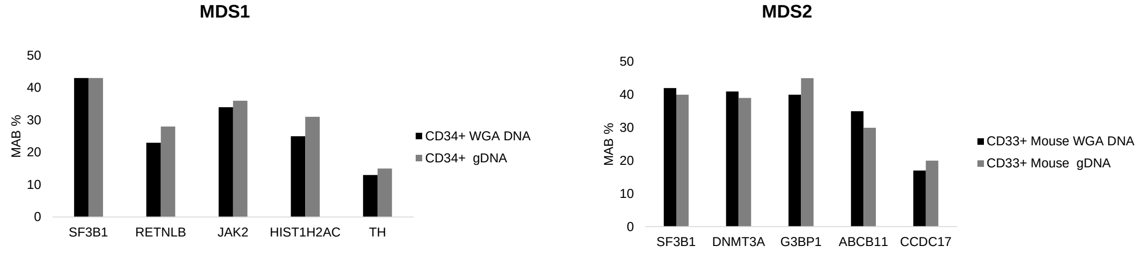

Supplementary Figure 4: Engraftment of hematopoietic cells in the bone marrow of NOD/SCID/IL2rγ$^{-/-}$ (NSG) mice.

(A) Percentage of human CD45^{+} cells in the bone marrow of NSG mice at 6 and 18-20 weeks after transplantation for MDS 5 patient.
(B) Immunohistochemistry staining for human CD45^{+} cells in the Xenograft bone marrow sample. Femurs were collected from engrafted and non-engrafted mice, and stained for human CD45 (in brown) and counterstained with Hematoxylin (in blue).

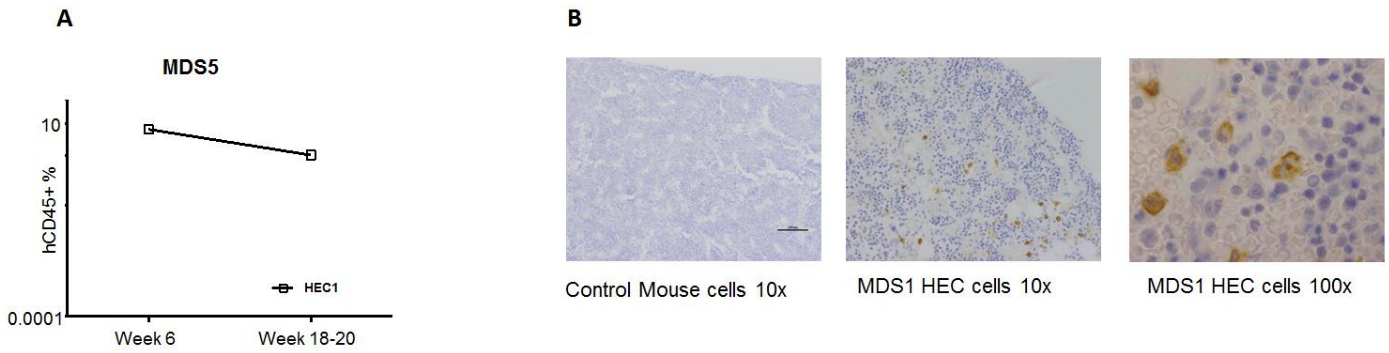

Supplementary Figure 5: Mutational architecture and sub-clonal evolution of *SF3B1* mutated MDS-RS bone marrow cells.

Panels show the gene mutations with their MABs in primary CD34^{+} bone marrow, HEC cells and LTC samples from each patient. MABs for HEC samples are the average between ≥1 mouse samples (where applicable). Three independent PCRs were performed to confirm/determine the MAB throughout the experiments. *- represents the significant changes in the MABs (t-test P <0.05). MAB-Mutant allele burden. HEC-Human engrafted cells. LTC-Long-term culture. BM- bone marrow. Mut- mutation.

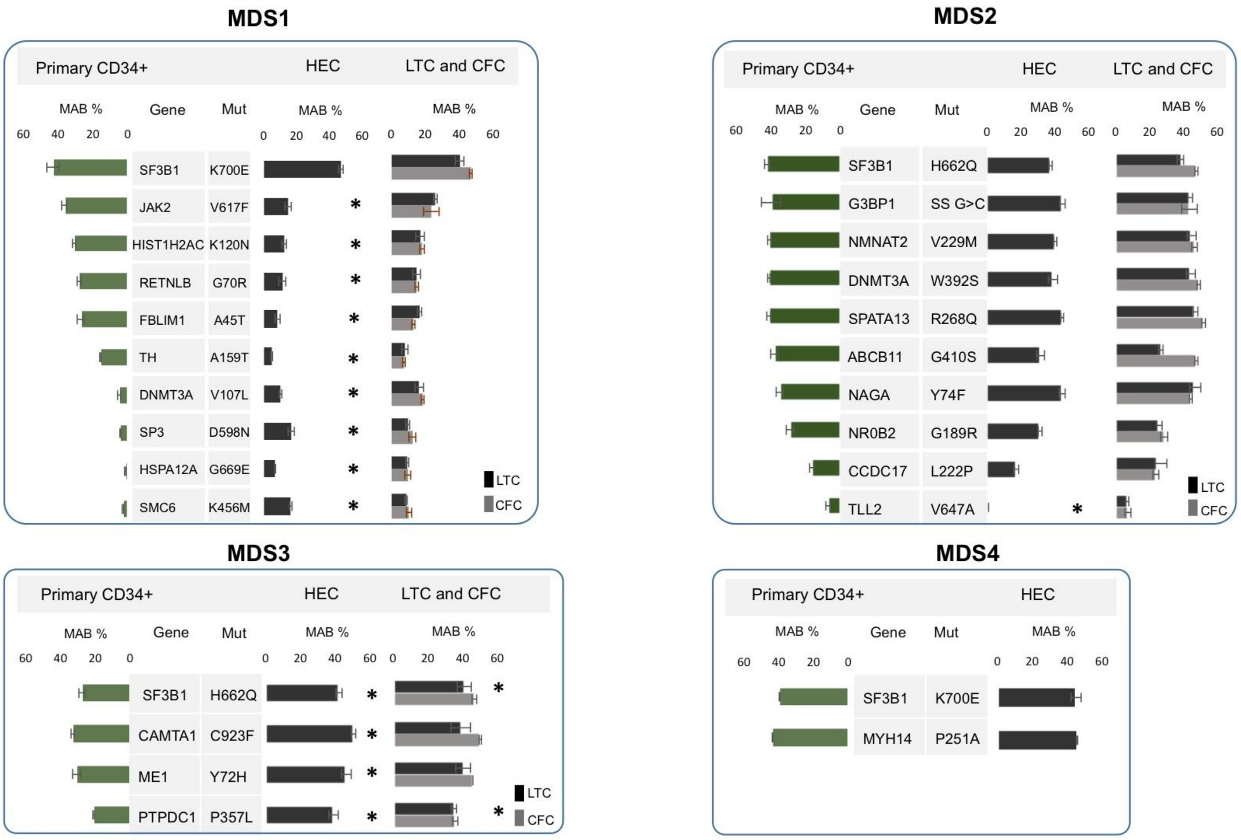

Supplementary Figure 6: *In-vitro* analysis of *SF3B1* mutant patient CD34+ cells.

Mutational analysis performed on BFU-E and CFU-GM colonies derived from primary CD34+ patient cells from MDS1. 96 individual colonies were screened for *JAK2* and *SF3B1* mutations. *SF3B1* mutations are seen equally in BFU-E and CFU-GM colonies.

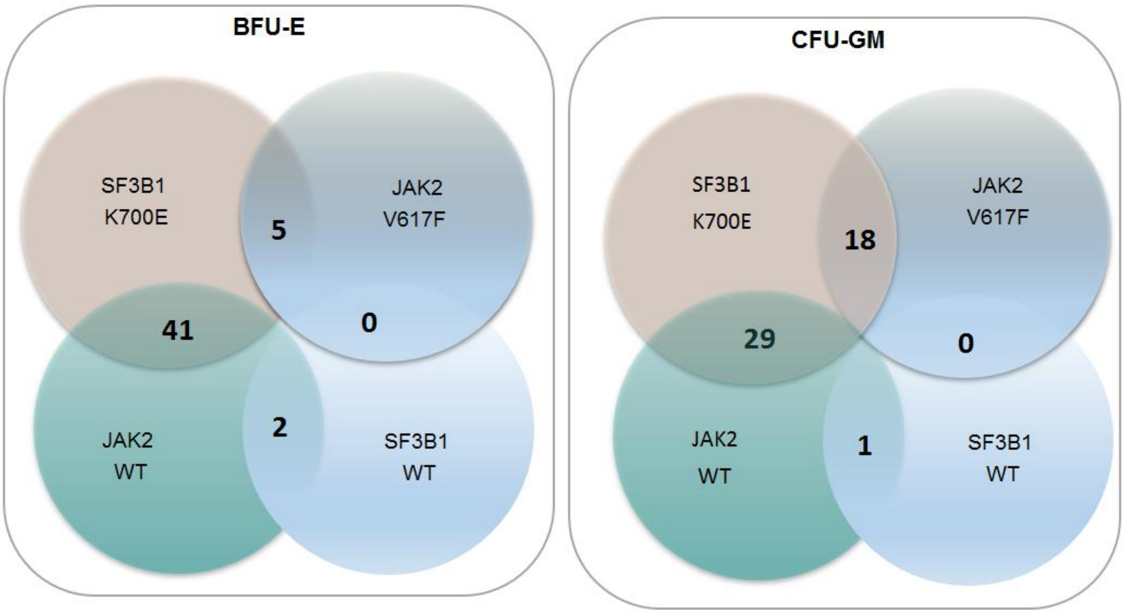

Supplementary Figure 7: Xenograft recapitulates the clonal changes occurring in the patient bone marrow compartment.

Mutational analysis of sequential primary TNC sample from MDS 2. Primary samples were received at two different time points, MDS 2 BM cells (TP1, MDS stage) and MDS 2 BM cells (TP3, AML Stage). Mutational analysis was also performed on human engrafted cells, which were obtained from mice transplanted with patient sample taken at time point 1. Three independent PCR/sequencing experiments were performed to confirm/determine the MAB throughout the experiments. MAB- Mutant allele burden. TP-Time point, HEC-human engrafted cells, BM- Bone marrow cells, ND- Not detected

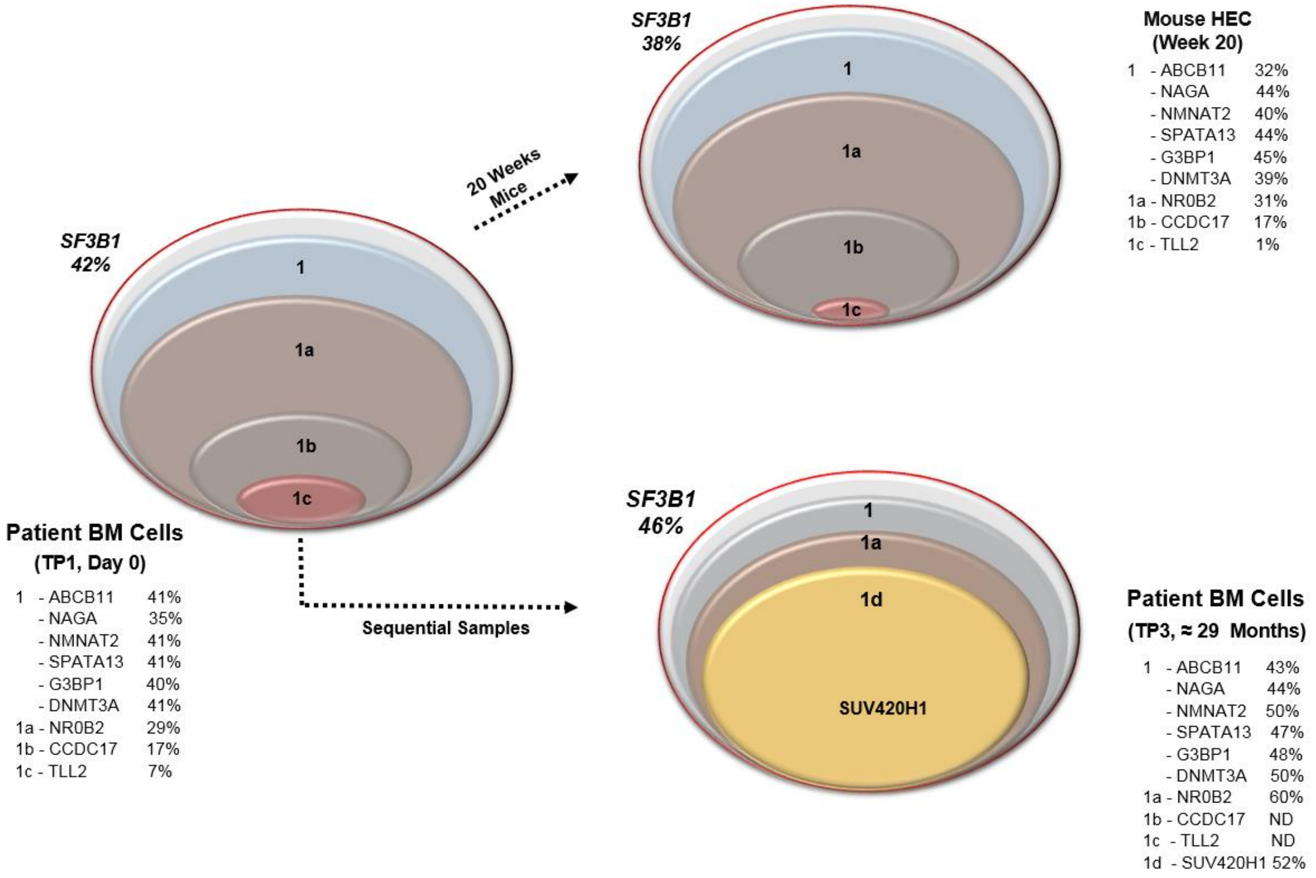

Supplementary Figure 8: **FISH, SNP (whole-exome) profiling and mutational analysis for MDS2**

(A) Detailed map of locus specific probe targeting q arm of chromosome 7. XL 7q22/7q36 probe detects a specific region at 7q22 (orange) including the *MLL5* gene while as the green-labelled probe hybridizes specifically to 7q36 (green) and includes the *EZH2* gene. In addition, a blue (aqua) labelled probe, which hybridizes to the centromeric region of chromosome 7 acts as a reference probe. Chr-Chromosome. (B) Single nucleotide variations (SNVs) profiles from whole-exome experiments based on $CD34^+$ cells (AML stage), paired constitutional DNA ($CD3^+$), $CD34^+$ (MDS stage time point1), Human engrafted cells (post engraftment, MDS stage time point 1) and LTC derived cells (MDS stage time point 1). SNVs were selected that were called germline or LOH in VarScan, due to presence at similar levels in both samples of a pair or significant allele burden change between the two respectively, had >20 reads depth. LOH- Loss of heterozygosity. (C) Mutational profile of sequential MLP sample for MDS2 obtained at AML stage of the disease. MAB- Mutant allele burden.

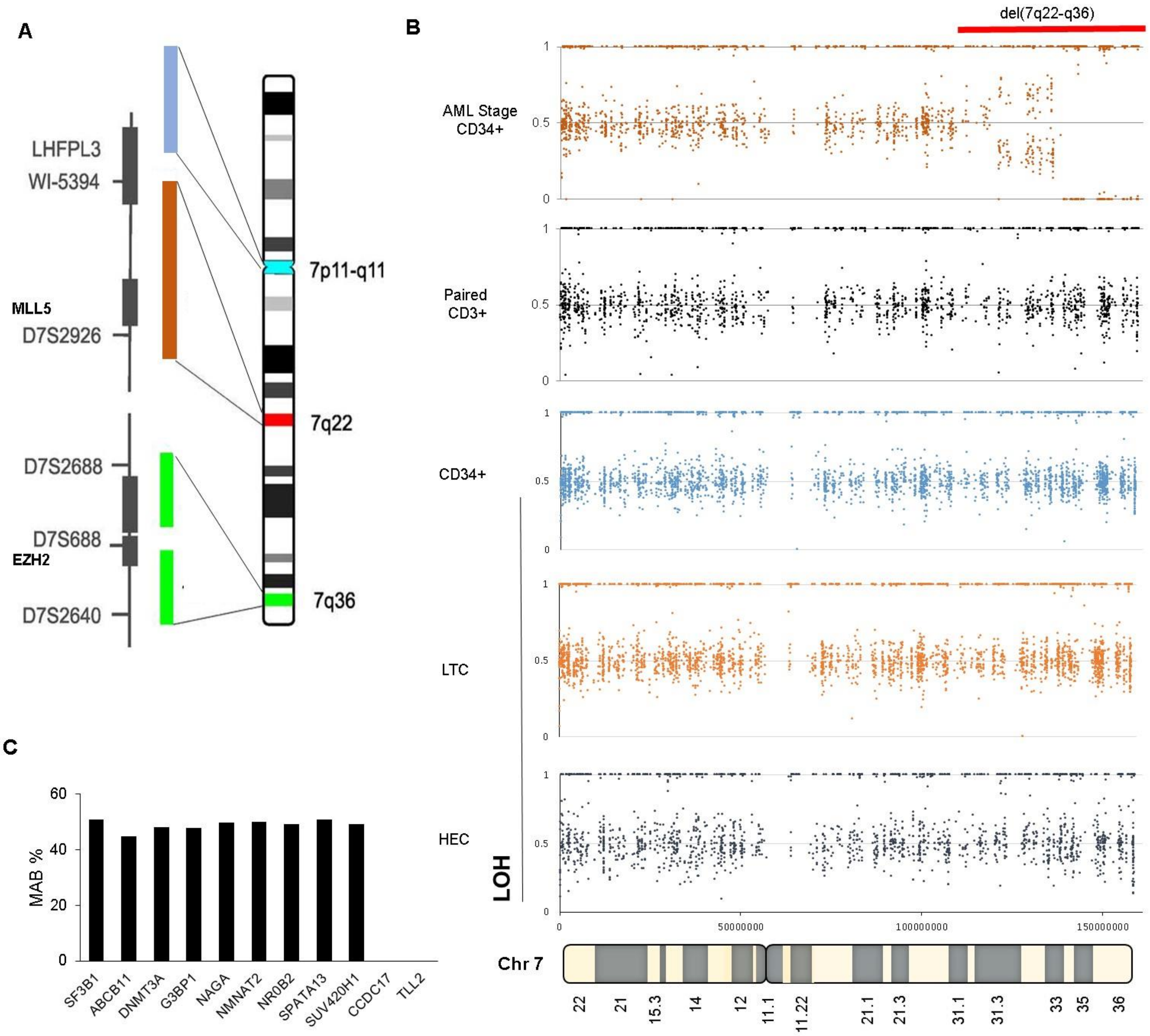

Supplementary Table 1. Detailed clinical characteristics of patients studied in this study.
* represent previously reported samples.

Patient ID	Sample Tim Point	Sample Time Point	Age	Sex	WHO Diagnosis	Treatment Prior To Sample	BM Blasts (%)	Hb	NPL	Platelet Counts	Ringed Sideroblast %	IPSS	Cytogenetics	Molecular Marker	Transfusion Dependant	Survival Status	AML Evolution	Disease Progression
**MDS 1**	TP1	1	77	M	RARS-T	Epo/GCSF	2	8.8	9.53	268	>15	Low	NORMAL	SF3B1 K700E	Yes	A	No	No
**MDS 1**	TP2	2	-		RARS-T	Epo/Len	2	10.5	1.9	248	>15	Low	NORMAL	-	Yes	-	No	No
**MDS 2**	TP1	1	75	M	RCMD-RS	Epo/GCSF	1	8.6	1.87	166	41	Low	NORMAL	SF3B1 H662Q	Yes	-	No	Yes
**MDS 2**	TP2	2	-	-	RCMD-RS	Epo/Len	1	7.1	3.67	137	50	-	NORMAL	-	Yes	-	No	Yes
**MDS 2**	TP3	3	-	-	sAML	Epo/Len	90	7.5	1.63	29	-	-	46,XY,add(7)(q32),add(10)(p13)[7]/ 46,XY,add(7)(q22),add(7)(q32),add(10)(p13)[3]	-	Yes	D	Yes	Yes
**MDS 3**	N/A	N/A	63	M	RARS	Epo/GCSF	1	9.1	3.15	570	>15	Low	NORMAL	SF3B1 H662Q	No	A	No	No
**MDS 4**	N/A	N/A	58	F	RARS	None	1	10.7	3.21	196	24	Low	NORMAL	SF3B1 K700E	No	A	No	No
**MDS 5**	N/A	N/A	22	M	Congenital sideroblastic anaemia	None	1	10.6	4.7	400	32	N/A	NORMAL	*ALAS2* R425C	Yes	A	No	No
**MDS 6 ***	N/A	N/A	74	M	RCMD-RS	Epo/Len/ Aza	1	12.1	3.44	151	35	Low	NORMAL	SF3B1 E622D	No	A	No	No
**MDS 14**	N/A	N/A	65	M	RARS	None	0	13	1.94	239	20	Low	NORMAL	SF3B1 H662Q	No	A	No	No
**MDS 7 ***	N/A	N/A	67	M	RARS	ATG	0	10.4	4.5	358	50	Low	NORMAL	SF3B1 H662Q	Yes	A	No	No
**MDS 8 ***	N/A	N/A	58	M	RARS	None	0	7.7	0.9	281	50	Int-I	NORMAL	SF3B1 H662Q	Yes	A	No	No
**MDS 9 ***	N/A	N/A	60	M	RARS	Epo/GCSF	1	9.8	5.3	334	47	Low	NORMAL	SF3B1 D781G	No	A	No	No
**MDS 10 ***	N/A	N/A	63	F	RARS-T	None	1	10.5	6	565	80	Low	NORMAL	SF3B1 E622D	No	A	No	No
**MDS 11 ***	N/A	N/A	73	M	RARS	Epo/GCSF/ ATO	0	9.7	2.80	236	78	Low	NORMAL	SF3B1 E622D	Yes	A	No	No
**MDS 12 ***	N/A	N/A	67	F	RARS	EPO/GCSF	2	7.1	1.7	389	>35	Int-I	NORMAL	SF3B1 K700E	Yes	A	No	No
**MDS 13 ***	N/A	N/A	60	M	tMDS	None	0	10.20	1.45	53	>25	Int-II	45,XY,t(1;6;8)(p32;p21;q12),-5,-7,+r [9]/46,XY [3]	TP53 Y220C	Yes	D	No	Yes

Abbreviations: M-male, F-female, A- alive, D-dead, BM- bone marrow, Hb-haemoglobin, Int1-intermediate, Int II-intermediate II, NA- not applicable. TP-time point. RARS- refractory anaemia with ringed sideroblast, RCMD-RS- refractory cytopenia with multilineage dysplasia and ringed sideroblasts, sAML-secondary AML, tMDS-therapy-related MDS, Len-lenalidomide, EPO-erythropoietin, GCSF-granulocyte-colony stimulating factor, *AZA*-azacitidine, ATG- anti-thymocyte globulin, ATO- arsenic trioxide

Supplementary Table 2. Detailed information for somatic mutations identified by whole-exome sequencing in 12/13 patients. Complete list of somatic mutation detected in patients with a paired constitutional DNA are shown in table. However, patient where no constitutional DNA was available (n=2), only known myeloid specific somatic mutations are shown in table. No acquired mutations were detected in congenital sideroblastic anaemia.

Patient ID	Gene	Ensembl_Protein_ID	Chromosome Location	Reference Base	Mutant Base	Codon Change	MAB (%)	Constitutional DNA Source	COSMIC	SIFT Score	SIFT Prediction	PolyPhen	Gene/Protein function
**MDS 1**	SF3B1	ENSP 00000335321	chr2: 198266834	T	C	K700E	43	Skin	yes	0	Damaging	Probably damaging	Subunit of the splicing factor SF3B required for 'A' complex assembly formed by the stable binding of U2 snRNP to the branch-point sequence (BPS) in pre-mRNA
**MDS 1**	HIST1H2AC	ENSP 00000321389	chr6 :26124820	G	T	K120N	31	Skin	no	0.01	* Damaging	Benign	Core component of nucleosome
**MDS 1**	FBLIM1	ENSP 00000364921	chr1: 16091611	G	A	A45T	27	Skin	no	0.35	Tolerated	Benign	Implicated in cell shape modulation (spreading) and motility
**MDS 1**	RETNLB	ENSP 00000295755	chr3: 108475355	C	T	G70R	28	Skin	no	0.03	Damaging	Probably damaging	Probable hormone
**MDS 1**	JAK2	ENSP 00000371067	chr9: 5073770	G	T	V617F	36	Skin	yes	0	Damaging	Probably damaging	Non-receptor tyrosine kinase involved in various processes such as cell growth, development, differentiation or histone modifications
**MDS 1**	TH	ENSP 00000370571	chr11: 2189733	C	T	A159T	15	Skin	no	0.02	Damaging	Benign	Plays an important role in the physiology of adrenergic neurons
**MDS 2**	SF3B1	ENSP 00000335321	chr2: 198267371	G	C	H662Q	42	CD3+ T-cells	yes	0.01	Damaging	Probably damaging	Subunit of the splicing factor SF3B required for 'A' complex assembly formed by the stable binding of U2 snRNP to the branch-point sequence (BPS) in pre-mRNA
**MDS 2**	G3BP1	ENSP 00000377681	chr5: 151175137	G	C	SS Exon 6 G>C	40	CD3+ T-cells	no	N/A	N/A	N/A	DNA-unwinding enzyme
**MDS 2**	DNMT3A	ENSP 00000370122	chr2: 25467133	C	G	W581S	41	CD3+ T-cells	no	1	Tolerated	Probably damaging	Required for genome-wide de novo methylation
**MDS 2**	TLL2	ENSP 00000350630	chr10: 98145885	A	G	V647A	7	CD3+ T-cells	no	0.15	Tolerated	Probably damaging	Protease which specifically processes pro-lysyl oxidase. Required for the embryonic development
**MDS 2**	NMNAT2	ENSP 00000294868	chr1: 183230405	C	T	V224M	41	CD3+ T-cells	no	0.02	Damaging	Probably damaging	Catalyses the formation of NAD(+) from nicotinamide mononucleotide (NMN) and ATP
**MDS 2**	NAGA	ENSP 00000379680	chr22: 42463872	T	A	Y74F	35	CD3+ T-cells	no	0.05	Damaging	Possibly damaging	Required for the breakdown of glycolipids
**MDS 2**	ABCB11	ENSP 00000263817	chr2: 169833167	C	T	G410S	38	CD3+ T-cells	no	0	Damaging	Probably damaging	Involved in the ATP-dependent secretion of bile salts into the canaliculus of hepatocytes
**MDS 2**	NR0B2	ENSP 00000254227	chr1: 27238545	C	T	G189R	29	CD3+ T-cells	no	0.03	Damaging	Benign	Acts as a transcriptional regulator
**MDS 2**	SPATA13	ENSP 00000371576	chr13: 24797870	G	A	R268Q	41	CD3+ T-cells	no	0.73	Tolerated	N/A	Acts as guanine nucleotide exchange factor (GEF) for RHOA, RAC1 and CDC42 GTPases
**MDS 2**	CCDC17	ENSP 00000389415	chr1: 46088498	A	G	L213P	17	CD3+ T-cells	no	0.29	Tolerated	Benign	Unknown
**MDS 3**	SF3B1	ENSP 00000335321	chr2: 198267371	G	T	H662Q	28	Skin	yes	0.01	Damaging	Probably damaging	Subunit of the splicing factor SF3B required for 'A' complex assembly formed by the stable binding of U2 snRNP to the branch-point sequence (BPS) in pre-mRNA

Patient ID	Gene	Ensembl_Protein_ID	Chromosome Location	Reference Base	Mutant Base	Codon Change	MAB (%)	Constitutional DNA Source	COSMIC	SIFT Score	SIFT Prediction	PolyPhen	Gene/Protein function
**MDS 3**	ME1	ENSP 00000358719	chr6: 84108234	A	G	Y72H	31	Skin	no	0.32	Tolerated	Probably damaging	Unknown
**MDS 3**	CAMTA1	ENSP 00000306522	chr1: 7731086	G	T	C923F	33	Skin	no	0.02	Damaging	Possibly damaging	Transcriptional activator. May act as a tumour suppressor
**MDS 3**	PTPDC1	ENSP 00000288976	chr9: 96860488	A	T	P357L	21	Skin	no	0.1	Tolerated	Benign	May play roles in cilia formation and/or maintenance
**MDS 4**	SF3B1	ENSP 00000335321	chr2: 198266834	T	C	K700E	40	Skin	yes	0	Damaging	Probably damaging	Subunit of the splicing factor SF3B required for 'A' complex assembly formed by the stable binding of U2 snRNP to the branch-point sequence (BPS) in pre-mRNA
**MDS 4**	MYH14	**ENST 00000262269**	chr19: 50728875	C	G	P251A	44	Skin	no	0	*Damaging	Probably damaging	Cellular myosin that appears to play a role in cytokinesis, cell shape, and specialized functions such as secretion and capping
**MDS 6**	*FGD1*	ENS P00000364277	chrX: 54492200	G	A	R476W	23	Skin	no	0	Damaging	Probably damaging	Activates CDC42, a member of the Ras-like family of Rho- and Rac proteins. Plays a role in regulating the actin cytoskeleton and cell shape.
**MDS 6**	*TESC*	ENSP 00000432716	chr12: 117479796	G	A	P148S	25	Skin	no	0.19	Tolerated	Probably damaging	Functions as an integral cofactor in cell pH regulation, cell maturation, cellular transport and cell surface stability.
**MDS 6**	*SF3B1*	ENSP 00000335321	chr2: 198267491	C	G	E622D	40	Skin	yes	0.05	Damaging	Possibly damaging	Subunit of the splicing factor SF3B required for 'A' complex assembly formed by the stable binding of U2 snRNP to the branch-point sequence (BPS) in pre-mRNA
**MDS 6**	***DNMT3A***	ENSP 00000264709	chr2: 25463286	C	T	R736H	8	CD3+ T-cells	yes	0.39	Tolerated	Probably damaging	Required for genome-wide de novo methylation
**MDS 7**	*ATP1A4*	ENSP 00000357060	chr1: 160143980	C	A	H691N	24	CD3+ T-cells	no	0	* Damaging	Possibly damaging	Part of ion pump responsible for maintaining sodium and potassium electrochemical gradients across the plasma membrane
**MDS 7**	*MCM3AP*	ENSP0000038 0820	chr21: 47666752	G	A	Q1447X	23	CD3+ T-cells	no	N/A	Tolerated	N/A	Involved in the nuclear localization pathway of MCM3
**MDS 7**	*SF3B1*	ENSP 00000335321	chr2: 198267371	G	C	H662Q	45	CD3+ T-cells	yes	0.01	Damaging	Probably damaging	Subunit of the splicing factor SF3B required for 'A' complex assembly formed by the stable binding of U2 snRNP to the branch-point sequence (BPS) in pre-mRNA
**MDS 7**	*GPR112*	ENSP 00000377699	chrX: 135455180	T	C	M2578T	25	CD3+ T-cells	no	0.34	Tolerated	Benign	Orphan receptor
**MDS 8**	*PKHD1*	ENSP 00000360158	chr6: 51947297	C	A	L58F	37	Skin	no	0.27	Tolerated	Probably damaging	May be required for correct bipolar cell division through the regulation of centrosome duplication and mitotic spindle assembly
**MDS 8**	*MC2R*	ENSP 00000333821	chr18: 13885474	G	A	A15V	63	Skin	no	0.05	Damaging	Benign	Member of the five-member G-protein associated melanocortin receptor family
**MDS 8**	*PCNT*	ENSP 00000352572	chr21: 47836271	A	G	I2147V	40	Skin	no	1	Tolerated	Benign	Integral component of the filamentous matrix of the centrosome involved in the initial establishment of organized microtubule arrays in both mitosis and meiosis
**MDS 8**	*SF3B1*	ENSP 00000335321	chr2: 198267371	G	C	H662Q	37	Skin	yes	0.01	Damaging	Probably damaging	Subunit of the splicing factor SF3B required for 'A' complex assembly formed by the stable binding of U2 snRNP to the branch-point sequence (BPS) in pre-mRNA
**MDS 8**	*ATG9B*	ENSP 00000475005	chr7: 150721444	C	T	G23R	44	Skin	no	N/A	N/A	Benign	Plays a role in the regulation of autophagy
**MDS 8**	*FITM2*	ENSP 00000380037	chr20: 42935567	C	T	A163T	48	Skin	yes	0.8	Tolerated	Benign	Plays an important role in lipid droplet accumulation. Plays a role in the regulation of cell morphology and cytoskeletal organization
**MDS 8**	*AFF3*	ENSP 00000386834	chr2: 100209883	G	T	P747H	19	Skin	no	0.09	Tolerated	Brobably damaging	Putative transcription activator that may function in lymphoid development and oncogenesis. Binds, in vitro, to double-stranded DNA
**MDS 8**	*TET2*	ENSP 00000442788	chr4:106164862	C T	-	1244_12 44del	45	Skin	no	0.03	Damaging	N/A	Dioxygenase that catalyses the conversion of the modified genomic base 5mC into 5hmC and plays a key role in active DNA demethylation
**MDS 8**	*CEP110*	ENSP 00000362972	chr9: 123860787	A	G	S249G	25	Skin	no	0.33	Tolerated	Probably damaging	Required for centrosome duplication at different stages of procentriole formation.
**MDS 9**	*AGXT2L1*	ENSP 00000296486	chr4: 109680914	G	A	T103I	37	Skin	no	0.08	Tolerated	benign	Catalyses the pyridoxal-phosphate-dependent breakdown of phosphoethanolamine
**MDS 9**	*IKBKE*	ENSP 00000356087	chr1: 206658341	A	G	S394G	29	Skin	no	0.41	Tolerated	Benign	Phosphorylates inhibitor of NF-kappa-B
**MDS 9**	*GAB4*	ENSP 00000383431	chr22: 17472858	C	T	R128H	40	Skin	yes	0	Damaging	Probably damaging	Unknown
**MDS 9**	*OC90*	ENSP 00000254627	chr8: 133036862	C	T	A434T	37	Skin	no	0.77	Tolerated	Benign	Unknown
**MDS 9**	*SF3B1*	ENSP 00000335321	chr2: 198266494	T	C	D781G	45	Skin	yes	0	Damaging	Probably damaging	Subunit of the splicing factor SF3B required for 'A' complex assembly formed by the stable binding of U2 snRNP to the branch-point sequence (BPS)

Patient ID	Gene	Ensembl_Protein_ID	Chromosome Location	Reference Base	Mutant Base	Codon Change	MAB (%)	Constitutional DNA Source	COSMIC	SIFT Score	SIFT Prediction	PolyPhen	Gene/Protein function
													in pre-mRNA
**MDS 10**	ANXA7	ENSP 00000362012	chr10: 75143371	C	-	T259fs	26	CD3+ T-cells	no	0	Damaging	N/A	Calcium/phospholipid-binding protein which promotes membrane fusion and is involved in exocytosis
**MDS 10**	SF3B1	ENSP 00000335321	chr2:198266834	T	C	K700E	40	CD3+ T-cells	yes	0	Damaging	Probably damaging	Subunit of the splicing factor SF3B required for 'A' complex assembly formed by the stable binding of U2 snRNP to the branch-point sequence (BPS) in pre-mRNA
**MDS 10**	DNMT3A	ENSP 00000264709	chr2:25457242	C	T	R882H	50	CD3+ T-cells	yes	0.03	Damaging	Possibly damaging	Required for genome-wide de novo methylation
**MDS 10**	SLC7A2	ENSP 00000419140	chr8:17417899	C	T	S494L	39	CD3+ T-cells	no	0.01	Damaging	Benign	Involved in the transport of the cationic amino acids
**MDS 11**	SF3B1	ENSP 00000335321	chr2: 198267491	C	A	E622D	60	N/A	yes	0.05	Damaging	Possibly damaging	Subunit of the splicing factor SF3B required for 'A' complex assembly formed by the stable binding of U2 snRNP to the branch-point sequence (BPS) in pre-mRNA
**MDS 11**	DNMT3A	ENSP 00000264709	chr2: 25457242	C	T	R882H	75	N/A	yes	0.03	Damaging	Possibly damaging	Required for genome-wide de novo methylation
**MDS 12**	*KIAA1109*	ENSP 00000264501	chr4: 123109050	G	A	G210R	40	CD3+ T-cells	no	0.01	Damaging	Probably damaging	Unknown
**MDS 12**	*SF3B1*	ENSP 00000335321	chr2: 198266834	T	C	K700E	43	CD3+ T-cells	yes	0	Damaging	Probably damaging	Subunit of the splicing factor SF3B required for 'A' complex assembly formed by the stable binding of U2 snRNP to the branch-point sequence (BPS) in pre-mRNA
**MDS 12**	*TET2*	ENSP 00000442788	chr4: 106157287	A C	-	730_730 del	42	CD3+ T-cells	no	0.02	Damaging	N/A	Dioxygenase that catalyses the conversion of the modified genomic base 5mC into 5hmC and plays a key role in active DNA demethylation
**MDS 12**	*PPM1D*	ENSP 00000306682	chr17: 58740624	A	-	Q510fs	42	CD3+ T-cells	no	0.04	Damaging	N/A	Required for the relief of p53-dependent checkpoint mediated cell cycle arrest
**MDS 12**	*DNMT3A*	ENSP 00000264709	chr2: 25466800	G	A	R635W	50	CD3+ T-cells	yes	0	Damaging	Probably damaging	Required for genome-wide de novo methylation
**MDS 13**	TP53	ENSP 00000269305	chr17: 7578190	A	G	Y220C	42	N/A	yes	0	Damaging	Probably damaging	Acts as a tumour suppressor
**MDS 13**	TP53	ENSP 00000269305	chr17: 7576853	G	T	Q331H	41	N/A	yes	0.23	Tolerated	Probably damaging	Acts as a tumour suppressor

Abbreviations: MAB- Mutant allele burden, N/A – Not available, D- Dead, A- Alive.

Supplementary Table 3: Showing the frequency of *SF3B1* mutations and additional coexisting mutations in MDS patients. * represents SF3B1 mutant patients without mutations of *TET2*, *DNMT3A* and *ASXL1*

	**Total *SF3B1* mutations**	**n=182**	**n=63**	**n=24**	**n=22**	**Total n=291**
**1**	SF3B1+TET2	28%	27%	25%	18%	26%
**2**	SF3B1+DNMT3A	18%	32%	21%	18%	21%
**3**	SF3B1+ASXL1	8%	6%	5%	18%	8%
	SF3B1 Without 1,2,3 *	>50%	35%	51%	50%	
		**Papaemmanuil, et al. 2013, Blood**	**Bejar, et al 2012, JCO**	**Mian, et al. 2013 Haematologica**	**Xu et al. 2014, PNAS**	

Supplementary Table 4: Patient characteristics and engraftment of bone marrow CD34^{+} cells obtained from MDS-RS patients, one patient with congenital sideroblastic anemia and three haematologically normal controls.

Patient No	Diagnosis	Known Disease Related Mutations	Percentage of Ringed Sideroblast	Cytogenetics	WES	Stem Cell Analysis	LTC	CFC	IBM CD34+ Cells Injected in NSG Mice	No. of Mice Injected/Engrafted	Percentage of Human Cell Engraftment (Week 6)			Percentage of Human Cell Engraftment at Termination (week 18-20)		
											CD45+	CD45+ CD19+	CD45+ CD33+	CD45+	CD45+ CD19+	CD45+ CD33+
MDS1	RARS-T	SF3B1 K700E	>15%	Normal	Yes	Yes	Yes	Yes	200,000	1/1	0.1	5.6	76.4	4.9	0	90
									200,000	1/1	1.1	19	75	3.6	0	96
									200,000	1/1	0.2	3.5	88	3.8	0	92
MDS2	RCMD-RS	SF3B1 H662Q	41%	Normal	Yes	Yes	Yes	Yes	67,000	1/3	0	0	0	0.1	5	95
MDS3	RARS	SF3B1 H662Q	>15%	Normal	Yes	No	Yes	Yes	100,000	1/1	1.7	64	19	1.1	0.2	97
									100,000	1/1	0.1	33	28	0.3	0	97
									100,000	1/1	1.4	53.2	26	2.4	0.2	95
MDS4	RARS	SF3B1 K700E	24%	Normal	Yes	Yes	Yes	No	65,000	1/2	0	0	0	0.1	0	83
MDS6	RCMD-RS	SF3B1 E622D	35%	Normal	Yes	Yes	No	No	N/A	N/A	N/A	N/A	N/A	N/A	N/A	N/A
MDS5	Congenital Sideroblastic anaemia	ALAS2 R452C	32%	Normal	Yes	No	No	No	100,000	1/1	5.35	1.36	93	0.5	28.3	61
									100,000	1/1	1	2.43	94	0.3	3.17	89.3
Normal BM1	Haematologically Normal	N/A	N/A	N/A	No	No	No	No	100,000	1/1	N/A	N/A	N/A	2.87	87.00	12.2
									100,000	1/1	N/A	N/A	N/A	7.75	94.00	5.80
									100,000	1/1	N/A	N/A	N/A	9.9	89.00	10.00
Normal BM2	Haematologically Normal	N/A	N/A	N/A	No	No	No	No	100,000	1/1	N/A	N/A	N/A	1.4	94.00	4.30
									100,000	1/1	N/A	N/A	N/A	3.3	96.00	3.00
									100,000	1/1	N/A	N/A	N/A	3.6	92.00	6.60
									100,000	1/1	N/A	N/A	N/A	2.2	95.00	5.00
Normal BM3	Haematologically Normal	N/A	N/A	N/A	No	No	No	No	100,000	1/1	N/A	N/A	N/A	9.3	83.00	16.00
									100,000	1/1	N/A	N/A	N/A	21.2	56.00	43.00
									100,000	1/1	N/A	N/A	N/A	3.5	80.00	20.00
									100,000	1/1	N/A	N/A	N/A	9.8	84.00	15.00

Abbreviations: N/A- Not applicable.

Supplementary Table 5: Table showing a complete list of somatic mutations in 4 MDS-RS patients studied for In-vivo and In-vitro experiments. Mutations present in paired primary CD34$^+$ cells, HEC samples and LTC samples for each case are shown. . All mutations were confirmed independently by sequencing from 3 independent PCR reactions from all experiments, which enabled us to quantify the mutant allele burden more accurately in all cases. The acquired nature of the mutations was also confirmed by their absence in paired constitutional DNA (Skin Biopsy or CD3$^+$ T-Cells). HEC- Human engrafted cells retrieved from mice. LTC- Long-term culture, N/A- Not applicable, MAB- Mutant Allele Burden, BM-bone marrow.

Experiment	Gene	Gene ID	Chromosome	Chromosome Position	Amino Acid Change	Primary BM MAB	HEC1 MAB	HEC2 MAB	HEC3 MAB	LTC MAB
**MDS1**	SF3B1	NM_012433	2	198266834	K700E	43%	46%	46%	50%	40%
**MDS1**	JAK2	NM_004972	9	5073770	V617F	36%	17%	10%	17%	26%
**MDS1**	DNMT3A	NM_153759	2	25470588	V107L	4%	10%	9%	12%	16%
**MDS1**	FBLIM1	NM_017556	1	16091611	A45T	27%	11%	6%	8%	17%
**MDS1**	HIST1H2AC	NM_003512	6	26124820	K120N	31%	14%	10%	14%	17%
**MDS1**	HSPA12A	NM_025015	10	118434314	G669E	2%	7%	7%	6%	9%
**MDS1**	RETNLB	NM_032579	3	108475355	G70R	28%	15%	7%	12%	15%
**MDS1**	SMC6	NM_024624	2	17897511	K456M	2%	18%	16%	16%	9%
**MDS1**	SP3	NM_001017371	2	174777831	D598N	4%	19%	12%	18%	10%
**MDS1**	TH	NM_000360	11	2189733	A159T	15%	5%	4%	5%	8%
**MDS2**	SF3B1	NM_012433	2	198267371	H662Q	42%	38%	N/A	N/A	39%
**MDS2**	DNMT3A	NM_175629	2	25467133	W581S	41%	39%	N/A	N/A	44%
**MDS2**	NAGA	NM_000262	22	42463872	Y74F	35%	44%	N/A	N/A	46%
**MDS2**	NMNAT2	NM_170706	1	183230405	V224M	41%	40%	N/A	N/A	45%
**MDS2**	NR0B2	NM_021969	1	27238545	G189R	29%	31%	N/A	N/A	25%
**MDS2**	SPATA13	NM_001166271	13	24797870	R268Q	41%	44%	N/A	N/A	47%
**MDS2**	TLL2	NM_012465	10	98145885	V647A	7%	1%	N/A	N/A	7%
**MDS2**	G3BP1	NM_005754	5	151175137	SS Exon 6 G>C	40%	45%	N/A	N/A	44%
**MDS2**	ABCB11	NM_003742	2	169833167	G410S	38%	32%	N/A	N/A	27%
**MDS2**	CCDC17	NM_001190182	1	46088498	L213P	17%	17%	N/A	N/A	24%
**MDS3**	SF3B1	NM_012433	2	198267371	H662Q	28%	45%	36%	46%	41%
**MDS3**	ME1	NM_002395	6	84108234	Y72H	31%	37%	48%	53%	40%
**MDS3**	CAMTA1	NM_015215	1	7731086	C923F	33%	50%	53%	50%	39%
**MDS3**	PTPDC1	NM_001253829	9	96860488	P357L	21%	39%	35%	44%	35%
**MDS4**	SF3B1	NM_012433	2	198266834	K700E	40%	47%	N/A	N/A	N/A
**MDS4**	MYH14	NM_001145809	19	50728875	P251A	44%	46%	N/A	N/A	N/A

Abbreviations: HEC- Human engrafted cells retrieved from mice. LTC- Long-term culture, N/A- Not applicable, MAB- Mutant Allele Burden, BM-bone marrow.

Note 1

SF3B1* mutant cells maintain their differentiation and proliferation potential *in-vitro

To evaluate the *ex-vivo* clonal growth potential of *SF3B1* mutant bone marrow $CD34^+$ cells from 3 patients (MDS1, TP1; MDS2, TP1; MDS3) were plated in methylcellulose to assess their colony-forming cell (CFC) abilities and grown in liquid culture (long-term culture, or LTC). No preferential bias towards any particular cell lineages in the short-term CFC culture was observed. Furthermore, *SF3B1* mutated cells also maintained their proliferation potential in the LTC conditions. To study the clonal landscape of the *SF3B1* mutant cells, we performed WES and/or targeted mutational analysis on LTC derived cells and pooled-CFC. We showed that all mutations observed in patients $CD34^+$ cells were present and detectable in both CFC and LTC, at a variable MAB frequency (Supplementary Fig. 5).

REFERENCES

1. Koboldt, D.C., Chen, K., Wylie, T., Larson, D.E., McLellan, M.D., Mardis, E.R., Weinstock, G.M., Wilson, R.K., and Ding, L. (2009). VarScan: variant detection in massively parallel sequencing of individual and pooled samples. Bioinformatics *25*, 2283-2285.
2. Koboldt, D.C., Zhang, Q., Larson, D.E., Shen, D., McLellan, M.D., Lin, L., Miller, C.A., Mardis, E.R., Ding, L., and Wilson, R.K. (2012). VarScan 2: somatic mutation and copy number alteration discovery in cancer by exome sequencing. Genome research *22*, 568-576.
3. Koressaar, T., and Remm, M. (2007). Enhancements and modifications of primer design program Primer3. Bioinformatics *23*, 1289-1291.
4. Li, H., and Durbin, R. (2009). Fast and accurate short read alignment with Burrows-Wheeler transform. Bioinformatics *25*, 1754-1760.
5. Li, H., Handsaker, B., Wysoker, A., Fennell, T., Ruan, J., Homer, N., Marth, G., Abecasis, G., Durbin, R., and Genome Project Data Processing, S. (2009). The Sequence Alignment/Map format and SAMtools. Bioinformatics *25*, 2078-2079.
6. McKenna, A., Hanna, M., Banks, E., Sivachenko, A., Cibulskis, K., Kernytsky, A., Garimella, K., Altshuler, D., Gabriel, S., Daly, M.*, et al.* (2010). The Genome Analysis Toolkit: a MapReduce framework for analyzing next-generation DNA sequencing data. Genome research *20*, 1297-1303.
7. Notta, F., Doulatov, S., Laurenti, E., Poeppl, A., Jurisica, I., and Dick, J.E. (2011). Isolation of single human hematopoietic stem cells capable of long-term multilineage engraftment. Science *333*, 218-221.
8. Wang, K., Li, M., and Hakonarson, H. (2010). ANNOVAR: functional annotation of genetic variants from high-throughput sequencing data. Nucleic acids research *38*, e164.
9. Ye, K., Schulz, M.H., Long, Q., Apweiler, R., and Ning, Z. (2009). Pindel: a pattern growth approach to detect break points of large deletions and medium sized insertions from paired-end short reads. Bioinformatics *25*, 2865-2871.
